# Nitric Oxide Circumvents Virus-Mediated Metabolic Regulation during Human Cytomegalovirus Infection

**DOI:** 10.1128/mBio.02630-20

**Published:** 2020-12-15

**Authors:** Rebekah L. Mokry, Megan L. Schumacher, Neil Hogg, Scott S. Terhune

**Affiliations:** a Department of Microbiology and Immunology, Medical College of Wisconsin, Milwaukee, Wisconsin, USA; b Department of Biophysics, Medical College of Wisconsin, Milwaukee, Wisconsin, USA; c Marquette University and Medical College of Wisconsin Department of Biomedical Engineering, Medical College of Wisconsin, Milwaukee, Wisconsin, USA; Stony Brook University

**Keywords:** cytomegalovirus, nitric oxide, metabolism, mitochondrial respiration, TCA cycle, glutaminolysis

## Abstract

Nitric oxide is a versatile and critical effector molecule that can modulate many cellular functions. Although recognized as a regulator of infections, the inhibitory mechanism of nitric oxide against human cytomegalovirus (HCMV) replication remains elusive. We demonstrate that nitric oxide attenuates viral replication by interfering with HCMV-mediated modulation of several cellular processes. Nitric oxide exposure reduced HCMV genome synthesis and infectious viral progeny with cell-type-dependent differences observed. Mitochondrial respiration was severely reduced in both uninfected and HCMV-infected cells during exposure with little impact on ATP levels indicating changes in cellular metabolism. Metabolomics identified significantly altered small molecules in multiple pathways during nitric oxide exposure including nucleotide biosynthesis, tricarboxylic acid (TCA) cycle, and glutamine metabolism. Glutathione metabolites were increased coinciding with a reduction in the glutathione precursor glutamine. This shift was accompanied by increased antioxidant enzymes. Glutamine deprivation mimicked defects in HCMV replication and mitochondrial respiration observed during nitric oxide exposure. These data suggest that nitric oxide limits glutaminolysis by shuttling glutamine to glutathione synthesis. In addition, lipid intermediates were severely altered, which likely contributes to the observed increase in defective viral particles. Nitric oxide disrupts multiple cellular processes, and we had limited success in rescuing replication defects by supplementing with metabolic intermediates. Our studies indicate that nitric oxide attenuation of HCMV is multifactorial with interference in viral manipulation of cellular metabolism playing a central role.

## Abstract (precis)

Human cytomegalovirus is a prevalent pathogen that can cause serious disease in patients with compromised immune systems, including transplant patients and during congenital infection. HCMV lytic replication likely occurs in localized sites of infection with immune cells infiltrating and releasing nitric oxide with other effector molecules.

## INTRODUCTION

Human cytomegalovirus (HCMV) is a prevalent pathogen that establishes lifelong infection (reviewed in reference 1). While typically asymptomatic in healthy individuals, HCMV can cause serious disease in immunocompromised populations, patients receiving immunosuppressant therapies, or those with a secondary viral infection. Additionally, HCMV is the leading cause of virus-mediated birth defects and can result in severe mental and cognitive impairment, microcephaly, and sensorineural hearing loss (2). HCMV can infect and replicate in many cell types such as fibroblasts and epithelial, smooth muscle, and neural progenitor cells, whereas hematopoietic progenitor cells and monocytes are the main sites of latency (3). This broad cellular tropism likely facilitates systemic spread and resulting pathogenesis.

HCMV is heavily reliant on modulation of host metabolism, specifically glycolysis and glutaminolysis, for efficient replication. HCMV induces glucose uptake, consumption, and glycolytic flux during infection (4–7). Inhibition of glycolysis results in attenuated viral DNA synthesis and virus production (8). Glycolysis likely contributes to efficient HCMV replication by producing ATP or NADH and supplying pyruvate for conversion to acetyl coenzyme A (acetyl-CoA) which is then shuttled to fatty acid synthesis (9). Glutamine metabolism is another major component for productive HCMV replication. Glutamine uptake and glutaminolysis are induced during infection through increased activity of the enzymes glutaminase and glutamate dehydrogenase (9, 10). It has been proposed that α-ketoglutarate produced during glutaminolysis supports increased activation of the tricarboxylic acid (TCA) cycle (5, 11, 12).

As with other viral infections, the innate immune response is active during HCMV infection, and several nonspecific antiviral molecules are released in defense against the virus, including type I and II interferons and nitric oxide (reviewed in reference 13). Nitric oxide is a hydrophobic diatomic free radical signaling molecule that can be produced through the activity of inducible nitric oxide synthase (NOS2) by innate immune cells during infection (14). While widely recognized for its role in vascular health, nitric oxide plays an important role in clearance of microbial infections. Nitric oxide has been demonstrated to be inhibitory toward both RNA and DNA viruses (reviewed in reference 15), including those that establish lifelong infection such as herpesviruses. Herpes simplex virus 1 (HSV-1) replication was demonstrated to be inhibited by exogenous nitric oxide exposure (16), yet nitric oxide is implicated in immunopathogenesis during HSV-1 infection (17–19). Nitric oxide inhibits HSV-2 replication *in vitro* and *in vivo* (20). Further, endogenous nitric oxide inhibits Epstein-Barr virus (EBV) replication and promotes EBV latency through suppression of an immediate early gene (21, 22). However, the mechanism by which nitric oxide inhibits these herpesviruses is unknown.

Nitric oxide has also been shown to influence cytomegalovirus (CMV) infections. A recent case study described a previously healthy adult male with NOS2 deficiency who succumbed to HCMV infection despite evidence of previous common viral infections (23). This individual was homozygous for a NOS2 variant with a truncation predicted to lack the domain required for nitric oxide formation. The case study emphasizes the importance of nitric oxide in controlling HCMV infection. NOS2-deficient mice infected with murine CMV are also more susceptible to lethal infection and have higher viral loads (24). In AIDS patients with HCMV coinfection, HCMV-infected glial cells expressing NOS2 are localized to the retina (25). Moreover, patients with HCMV retinitis have elevated levels of nitric oxide in the aqueous humor compared to those without HCMV retinitis, and this was reduced after antiviral treatment (26). *In vitro* studies using a nitric oxide donor diethylamine NONOate (DEA/NO) during infection of retinal pigment epithelial cells observed reduced early and late protein expression (27). HCMV infection of endothelial and smooth muscle cells induces NOS2 mRNA in an IE2-dependent manner (28). More recent work by Nukui et al. (29) provides insight into potential mechanisms by identifying S-nitrosation, a modification involving nitric oxide, of several HCMV proteins including pp71, which disrupts its activity against the STING pathway. In contrast to lytic infection, nitric oxide produced during latent HCMV infection via NOS2 was demonstrated to be essential for viral latency by silencing of immediate early gene expression through an unknown mechanism (30). Although these studies demonstrate that nitric oxide is inhibitory to CMV, the overall effect of nitric oxide on HCMV replication and, more importantly, the inhibitory mechanism remain unknown.

Nitric oxide signaling is mediated through its inherent biological chemistry that can be largely explained by its rapid reactivity with other free radicals and some metal centers that result in posttranslational modifications or the production of secondary signals. Specifically, nitric oxide has high affinity for protein-bound ferrous iron. High levels of nitric oxide result in inhibition of enzymes containing ferrous heme groups and/or iron/sulfur clusters in their catalytic sites (reviewed in references 31 and 32). These enzymes include complex I and complex IV of the electron transport chain (ETC) (33–35), resulting in inhibition of respiration. Ribonucleotide reductase, which contains a stable tyrosyl radical in close proximity to the bi-iron center, has also been demonstrated to be inhibited by nitric oxide through rapid reaction with the tyrosyl radical, resulting in decreased DNA replication (36, 37). Further, nitric oxide can disrupt the TCA cycle and cellular metabolism through inhibition of the [4Fe-4S] cluster-containing aconitase, an enzyme involved in the TCA cycle (34). The impact of nitric oxide on these processes during HCMV infection is unknown.

In these studies, we sought to determine the mechanism of inhibition occurring during nitric oxide exposure using a slow-release nitric oxide donor. We observe that a physiological concentration of nitric oxide delays the onset of genome synthesis, suppresses viral gene expression, and reduces infectious virus production, and this occurs in a cell-type-dependent manner. We demonstrate that nitric oxide inhibits mitochondrial respiration and alters host metabolism. Altered glutamine metabolism, nucleotide biosynthesis, and lipid metabolism were observed with limited ability to rescue HCMV infection by supplementing with key metabolites during exposure. These studies define the pleiotropic effects of the innate immune effector nitric oxide on critical cellular processes required for HCMV infection.

## RESULTS

### Nitric oxide attenuates HCMV genome replication and reduces infectious virus production.

**FIG 1:**
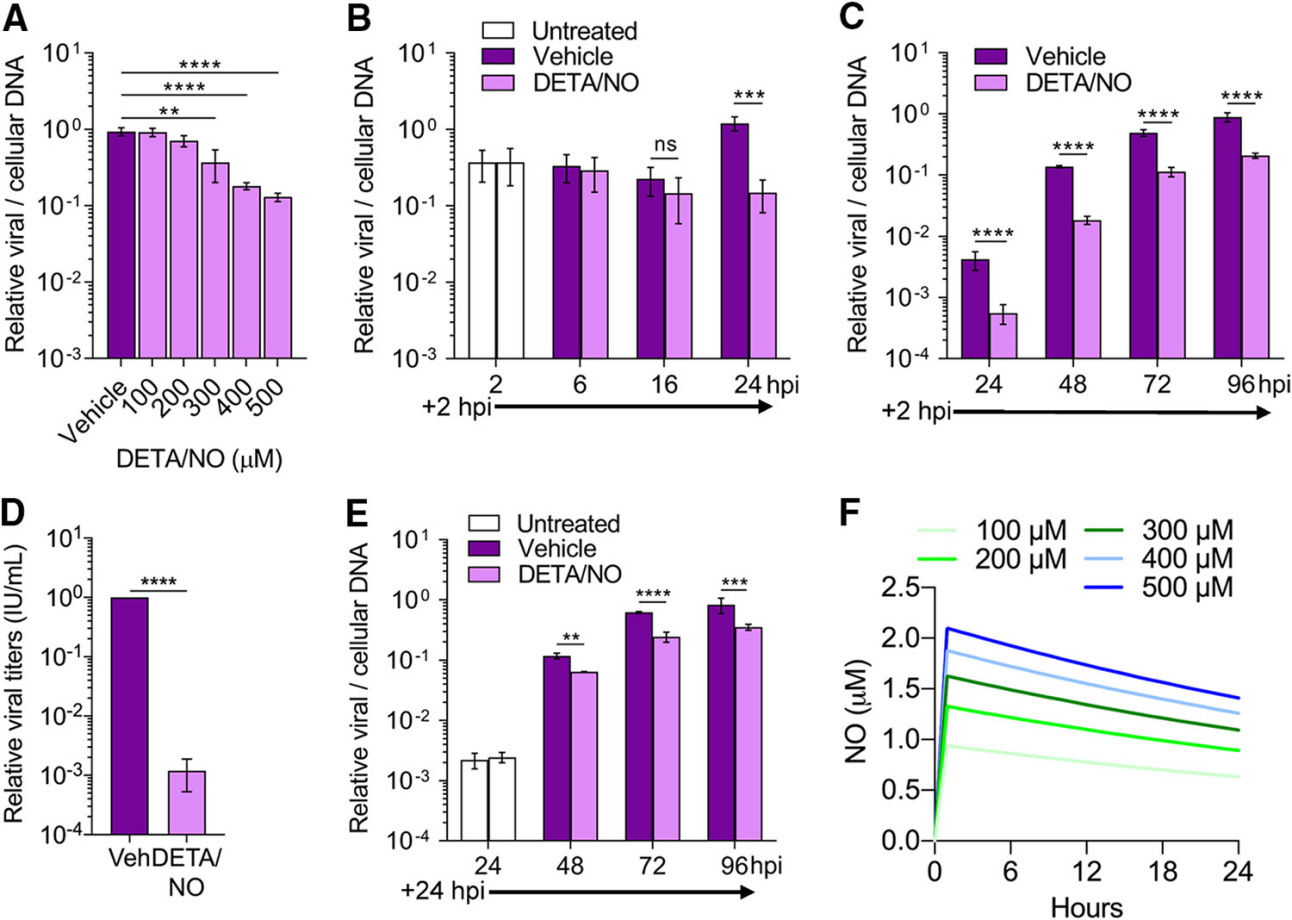
Physiological levels of nitric oxide attenuate HCMV genome replication and reduce infectious virion production from MRC-5 fibroblasts. (A) MRC-5 cells were plated at subconfluence, serum starved for 24 h, and infected with HCMV strain TB40/E encoding GFP (TB40/E-GFP) at an MOI of 3 infectious units/cell (IU/cell). Cells were treated at 2 hpi with the indicated concentrations of the nitric oxide donor diethylenetriamine NONOate (DETA/NO) or vehicle control. Relative viral to cellular DNA levels were measured at 24 hpi using quantitative PCR with primers to HCMV UL123 and cellular TP53 genes. *P *= 0.002, **; *P* < 0.0001, ****. (B to D) MRC-5 cells were infected as described above and treated at 2 hpi and every 24 h with 500 μM DETA/NO. Relative viral to cellular DNA levels were determined at the indicated time points (B and C), and viral titers were quantified by a TCID_50_ assay using culture supernatant collected at 96 hpi (D). (E) MRC-5 cells were infected as described above and treated starting at 24 hpi with 500 μM DETA/NO, and relative viral to cellular DNA levels were determined. *P* > 0.05, ns; *P* = 0.004, **; *P* = 0.0002, ***; *P* < 0.0001, ****. Data are the results of three biological replicates and two technical replicates. Error bars represent standard deviation from the mean. Statistical analyses were performed on transformed data for panels A to C and E. One-way ANOVA (A), two-way ANOVA (B, C, and E), or paired *t* test (D) was used to determine significance. (F) Estimated steady-state nitric oxide levels from various concentrations of DETA/NO were modeled using COPASI software. DETA/NO rate of decay was calculated using the *t*_1/2_ at 37°C (20 h). A fixed concentration of oxygen was estimated to be 220 μM, and the rate of nitric oxide and oxygen forming NO_2_ was set at 2.4 × 10^6^ M^−2^ s^−1^ (41). The rate of nitric oxide with NO_2_ forming nitrite was set at 1 × 10^9^ M^−2^ s^−1^.

Immune cells produce high levels of cytokines, and subsequently nitric oxide, in response to viral infection. Nitric oxide is primarily produced by these cells via NOS2. We sought to elucidate the antiviral mechanism of nitric oxide on HCMV replication using a spontaneous-release nitric oxide donor, diethylenetriamine NONOate (DETA/NO). This exogenous source of nitric oxide has a half-life of approximately 20 h at 37°C and releases 2 mol of nitric oxide for every mole of the parent compound leaving the diethylenetriamine backbone (38). Sustained nitric oxide release mimics immune cells producing nitric oxide during viral infection (39). To determine the impact of nitric oxide on HCMV replication in MRC-5 fibroblasts (Fig. 1), we infected subconfluent cells using HCMV strain TB40/E encoding green fluorescent protein (TB40/E-GFP) at a multiplicity of infection (MOI) of 3 infectious units per cell (IU/cell). Cells were treated at 2 h postinfection (hpi) with a range of 100 to 500 μM DETA/NO. Viral DNA levels were measured at 24 hpi using quantitative PCR (qPCR) (Fig. 1A). DETA/NO concentrations of 100 and 200 μM resulted in viral DNA levels similar to vehicle control. In contrast, concentrations of 300, 400, and 500 μM resulted in a respective 0.4-, 0.7-, and 0.9-log reduction in viral DNA levels (Fig. 1A). These data indicate that nitric oxide is inhibitory to HCMV viral DNA synthesis in a concentration-dependent manner.

Using 500 μM DETA/NO, we characterized the effect of nitric oxide throughout the replication cycle. Cells were treated starting at 2 hpi and retreated every 24 h. Viral DNA levels were measured at 2 to 24 hpi (Fig. 1B) and 24 to 96 hpi (Fig. 1C), and cell-free viral titers were quantified at 96 hpi (Fig. 1D). DETA/NO exposure had no effect on viral DNA levels prior to 24 hpi but again resulted in a 0.9-log reduction at 24 hpi (Fig. 1B). DNA levels at 48, 72, and 96 hpi were reduced by 0.9, 0.6, and 0.6 log, respectively, in DETA/NO-treated groups (Fig. 1C). Viral titers were decreased by 2.9 logs at 96 hpi (Fig. 1D) with a mean of 4.5 × 10^5^ IU/ml in vehicle control compared to 6.14 × 10^2^ IU/ml during DETA/NO treatment. Finally, to determine if nitric oxide has an effect after the onset of viral DNA synthesis, we delayed the addition of DETA/NO treatment to 24 hpi (Fig. 1E). We quantified 0.2-, 0.4-, and 0.3-log reductions at 48, 72, and 96 hpi, respectively, indicating DETA/NO has a greater impact on viral DNA synthesis if present before its onset. Together, these data demonstrate that nitric oxide exposure attenuates viral genome synthesis and infectious virus production, and the effect on viral DNA synthesis is influenced by the timing of exposure.

Physiological concentrations of nitric oxide during infection remain controversial but likely range from nanomolar to low micromolar depending on immune cells’ proximity (40). To estimate the nitric oxide exposure, we modeled nitric oxide release from DETA/NO using COPASI application software (Fig. 1F). We calculated the first-order rate constant for the decay of DETA/NO from the donor half-life (*t*_1/2_) at 37°C (9.627 × 10^−6^ s^−1^) and estimated a fixed oxygen concentration of 220 μM. The reaction rate of nitric oxide with oxygen to form NO_2_ is 2.4 × 10^6^ M^−2^ s^−1^ (41), while the reaction rate of nitric oxide with NO_2_ to form nitrite was estimated as 1 × 10^9^ M^−2^ s^−1^. The maximum predicted steady-state nitric oxide levels from 100 to 500 μM DETA/NO ranged from 1 to 2 μM (Fig. 1F), assuming oxygen levels remain constant. Consequently, the use of 500 μM DETA/NO is predicted to result in a sustained concentration of nitric oxide that is within the low-micromolar levels predicted during viral infection.

### Nitric oxide release from DETA/NO, but not nitric oxide oxidation products, attenuates replication.

**FIG 2:**
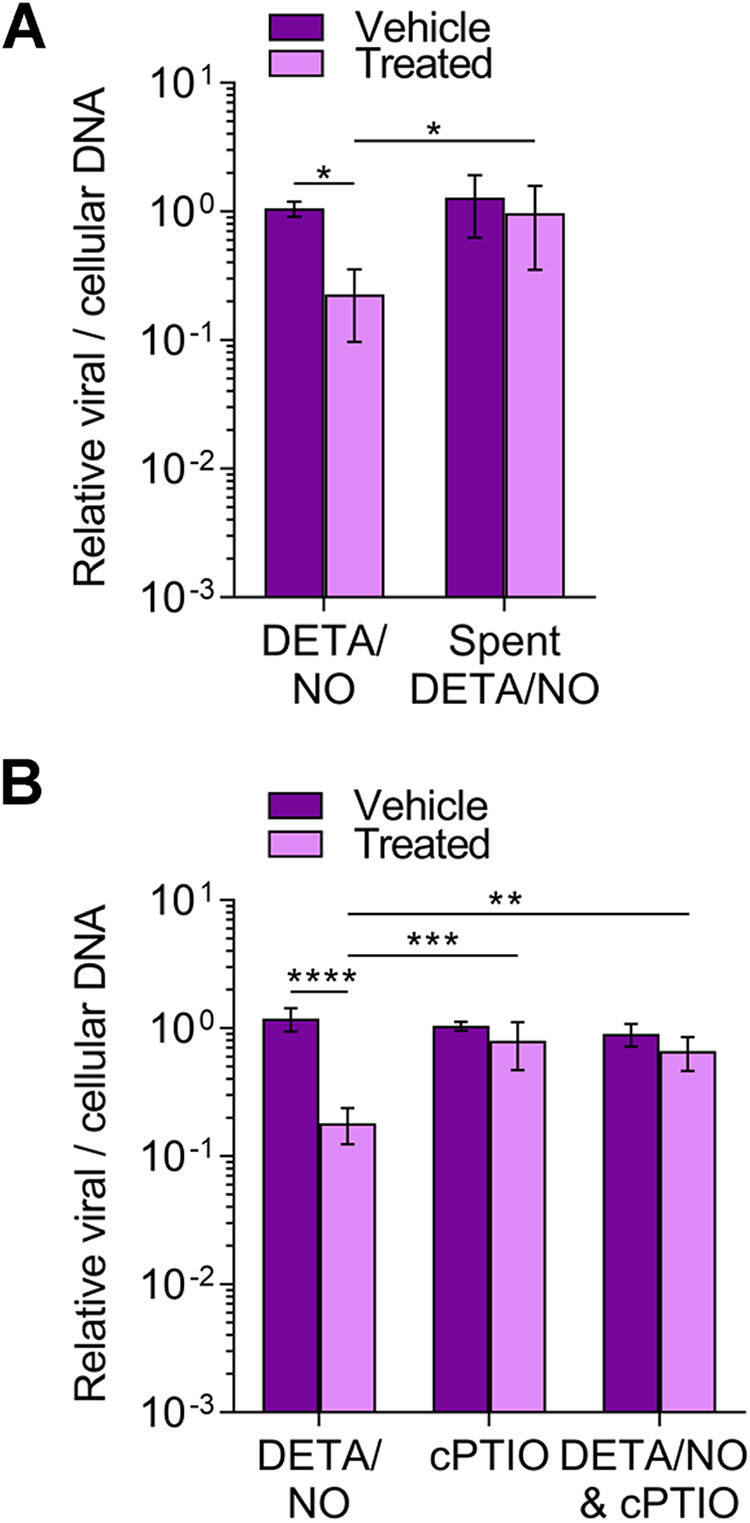
Nitric oxide release from DETA/NO but not nitric oxide oxidation products attenuates viral genome replication. MRC-5 cells were plated at subconfluence, serum starved for 24 h, and infected with TB40/E-GFP at an MOI of 3 IU/cell. Cells were treated at 2 hpi with 500 μM DETA/NO, spent DETA/NO (A), nitric oxide scavenger cPTIO (B), or vehicle control. Cells were collected at 24 hpi, and relative viral to cellular DNA levels were determined by quantitative PCR using primers to HCMV UL123 and cellular TP53 genes. Data are the results of three biological replicates and two technical replicates. Error bars represent standard deviation from the mean. Statistical analyses were performed on transformed data, and two-way ANOVA was used to determine significance. *P* < 0.05, *; *P* = 0.001, **; *P* = 0.0005, ***; *P* < 0.0001, ****.

The release of nitric oxide from DETA/NO results in oxidation products such as nitrite and nitrate in addition to the backbone diethyltriamine (38). To determine if nitric oxide release from DETA/NO is solely responsible for the inhibition, we treated cells with spent DETA/NO that contains only the parental backbone molecule and oxidation products. Spent donor was prepared by incubating medium containing 500 μM DETA/NO for 48 h at 37°C. Fibroblasts were infected as previously described and treated at 2 hpi with 500 μM DETA/NO, spent DETA/NO, or vehicle control. At 24 hpi, viral DNA levels were measured by qPCR (Fig. 2A). Treatment with spent DETA/NO did not alter viral DNA levels, indicating oxidation products and DETA have limited effect on DNA synthesis. We confirmed that nitric oxide was acting to inhibit DNA synthesis by treating with a nitric oxide scavenger, cPTIO (2-4-carboxyphenyl-4,4,5,5-tetramethylimidazoline-1-oxyl-3-oxide) (Fig. 2B). cPTIO reacts with nitric oxide to form nitrogen dioxide and ultimately nitrite (42). We infected cells as described above and treated them at 2 hpi with 500 μM DETA/NO and/or 500 μM cPTIO. Viral DNA levels were restored at 24 hpi when treating with cPTIO and DETA/NO (Fig. 2B). Collectively, these data demonstrate that nitric oxide release from DETA/NO, and not nitric oxide oxidation products or DETA, is responsible for attenuated viral replication.

### Infected epithelial cells exhibit altered HCMV replication characteristics compared to fibroblasts after nitric oxide exposure.

**FIG 3:**
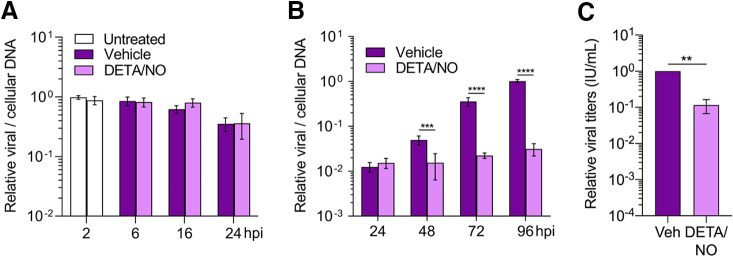
Infected ARPE-19 epithelial cells exhibit altered HCMV replication characteristics compared to fibroblasts after nitric oxide exposure. Confluent ARPE-19 cells were infected with HCMV strain TB40/E-GFP at an MOI of 5 IU/cell. At 2 hpi, cells were treated with 500 μM DETA/NO or vehicle control. Relative viral to cellular DNA levels were determined at the indicated time points using primers to HCMV UL123 and cellular TP53 genes (A and B), and viral titers were quantified by a TCID_50_ assay on MRC-5 cells using culture supernatant collected at 96 hpi (C). Data are the results of three biological replicates and two technical replicates. Statistical analyses were performed on transformed data for panel B. Two-way ANOVA with multiple comparisons (B) and paired *t* test (C) were used to determine significance. *P* = 0.001, **; *P* = 0.0002, ***; *P* < 0.0001, ****.

HCMV infects diverse cell types within the body. We explored the effect of nitric oxide in retinal pigment epithelial cells (ARPE-19) which are susceptible to infection and near sites of high nitric oxide production *in vivo*. Confluent ARPE-19 cells were infected at an MOI of 5 IU/cell. Cells were treated at 2 hpi and every 24 h with 500 μM DETA/NO, and viral DNA levels were assessed using qPCR (Fig. 3A and B). We observed a 0.5-log reduction in viral DNA levels beginning at 48 hpi (Fig. 3B). This decrease became more pronounced at 72 and 96 hpi with 1.2- and 1.5-log reductions, respectively. Unlike in fibroblasts, viral DNA levels exhibit little to no increase over time in treated epithelial cells compared to control. These results demonstrate that nitric oxide exposure has a greater impact on viral DNA synthesis in epithelial cells than in fibroblasts. However, we cannot rule out possible differences in HCMV replication kinetics between cell types contributing to the observed differences. We next quantified viral titers at 96 hpi. Viral titers were decreased by only 1 log (Fig. 3C) with a mean of 1.173 × 10^3^ IU/ml for vehicle control and 1.3 × 10^2^ IU/ml during nitric oxide exposure, despite the 1.5-log reduction of viral DNA. This result is in contrast with the 0.6-log reduction in viral DNA and 2.9-log reduction in viral titers at 96 hpi in fibroblasts (Fig. 1C and D). These data show that nitric oxide has a differential effect on HCMV replication between ARPE-19 epithelial cells and MRC-5 fibroblasts, ultimately affecting their ability to produce infectious virus by 96 hpi.

### Nitric oxide promotes cytostasis during exposure.

**FIG 4:**
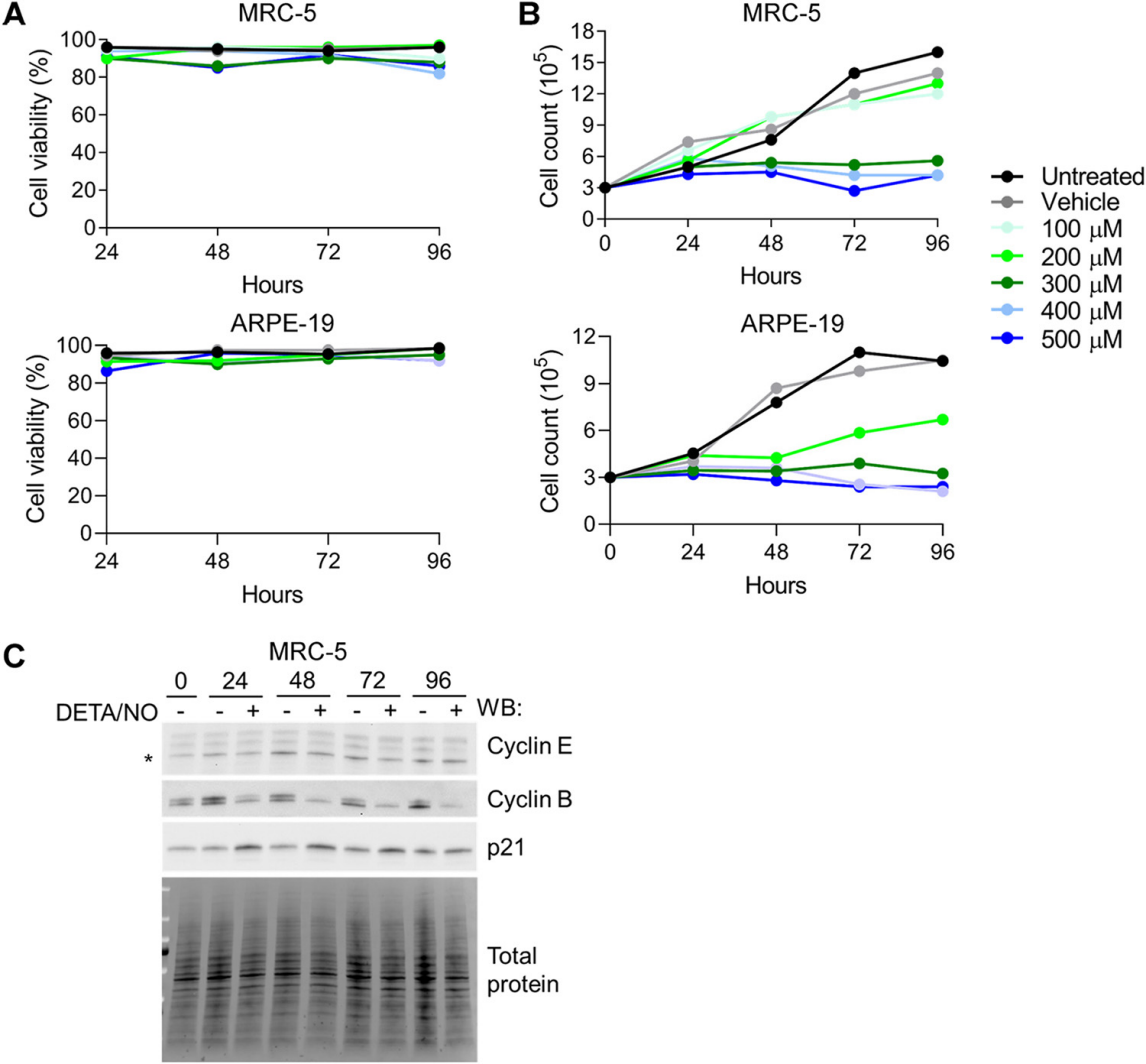
Nitric oxide promotes cytostasis and disrupts expression of cell cycle regulators. (A and B) Uninfected MRC-5 and ARPE-19 cells were plated at subconfluence and treated every 24 h with DETA/NO or vehicle control. Cell viability and live cell number were quantified at the indicated time points using an automated cell counter and trypan blue exclusion. Data represent two biological replicates. (C) MRC-5 cells were plated at subconfluence and treated every 24 h with 500 μM DETA/NO or vehicle control. Whole-cell lysates were collected at the indicated time points and analyzed by Western blotting using antibodies against the indicated cellular proteins. The asterisk (*) denotes the band for cyclin E. Total protein was used as a loading control. Data represent two biological replicates.

Nitric oxide has been demonstrated to have cytotoxic effects on different cell types (43–46). Therefore, we assessed the cytotoxicity of DETA/NO in uninfected fibroblasts and epithelial cells (Fig. 4A). Cells were plated subconfluently and treated with a range of 100 to 500 μM DETA/NO, vehicle control, or no treatment, and cell viability was assessed using trypan blue exclusion. Both cell types remained viable up to 96 h posttreatment, which was the latest time point tested, indicating that these concentrations of nitric oxide are not cytotoxic.

Nitric oxide can promote cellular proliferation at low levels but exhibits cytostatic effects at high concentrations (reviewed in reference 47). HCMV replication is influenced by dynamic changes in cell cycle regulators during infection. To investigate the possibility of nitric oxide-induced cytostasis, we treated cells as described above and quantified the number of live cells over time. Cell numbers did not increase over time when exposed to 300 to 500 μM DETA/NO (Fig. 4B). Epithelial cells showed similar cytostatic effects beginning at 200 μM DETA/NO (Fig. 4B). These higher concentrations attenuate viral DNA synthesis at 24 hpi and are cytostatic, suggesting that nitric oxide is modulating a cellular function that contributes to viral DNA synthesis. To further investigate nitric oxide-induced cytostasis, we examined levels of cell cycle regulators cyclin B, cyclin E, and p21^(CIP1/WAF1)^ in fibroblasts whose levels change during HCMV infection. During nitric oxide exposure of uninfected cells, cyclin B levels decreased over time relative to control (Fig. 4C) while cyclin E levels were relatively unchanged. In contrast, we observed increasing levels of the cyclin-dependent kinase (CDK) inhibitor p21. Collectively, these data indicate that higher concentrations of DETA/NO have a cytostatic or antiproliferative effect including altered expression of cell cycle regulators.

### Effects of nitric oxide on viral gene expression between cell types.

**FIG 5:**
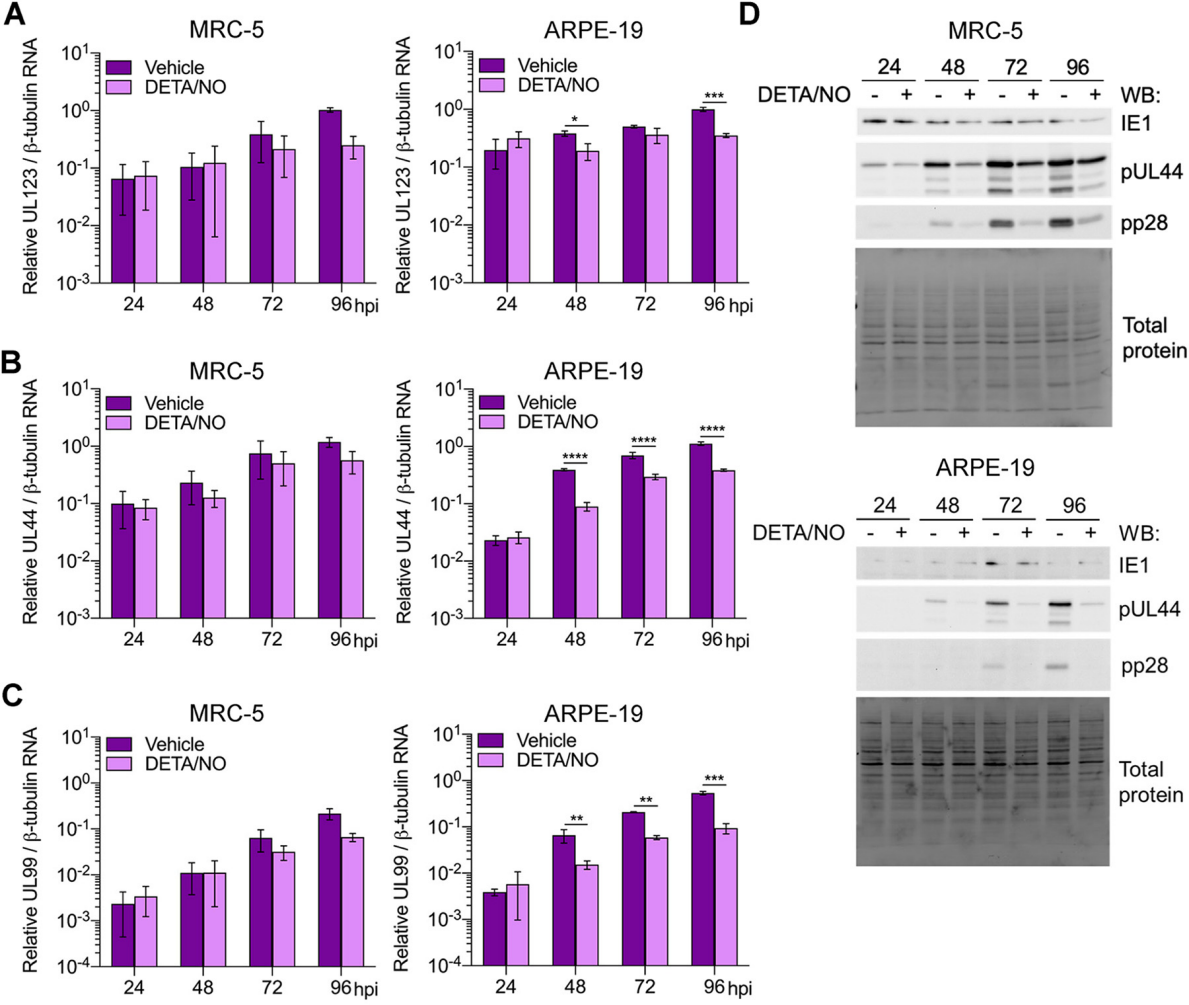
Differential effects of nitric oxide on viral gene expression between cell types. (A to C) MRC-5 cells were plated at subconfluence, serum starved for 24 h, and infected with HCMV strain TB40/E-GFP at an MOI of 3 IU/cell. Confluent ARPE-19 cells were infected at an MOI of 5 IU/cell. MRC-5 and ARPE-19 cells were treated at 2 hpi and every 24 h with 500 μM DETA/NO or vehicle control. Total RNA was isolated at the indicated time points, and HCMV UL123 (A), UL44 (B), and UL99 (C) levels relative to cellular β-tubulin RNA levels were determined by quantitative RT-PCR using gene-specific primers. Data are the results of three biological replicates and two technical replicates. Statistical analyses were performed on transformed data, and two-way ANOVA with multiple comparisons was used to determine significance. *P* = 0.02, *; *P* = 0.001, **; *P* < 0.001, ***; *P* < 0.0001, ****. (D) MRC-5 and ARPE-19 cells were infected and treated as described above. Whole-cell lysates were collected at the indicated time points (hours postinfection, above blot). Lysates were analyzed by Western blotting using antibodies against the indicated viral proteins. Total protein was used as a loading control. Data represent three biological replicates.

Viral DNA synthesis requires HCMV immediate early and early gene expression. Next, we quantified viral RNAs in infected fibroblasts and epithelial cells during nitric oxide exposure (Fig. 5). RNA levels in fibroblasts were similar between treated and control cells until 96 hpi. At this time point, HCMV UL123 (IE gene) (Fig. 5A), UL44 (E gene) (Fig. 5B), and UL99 (L gene) (Fig. 5C) were reduced but did not reach statistical significance. In contrast, in epithelial cells, RNA levels were decreased at multiple time points. HCMV UL123 levels were reduced beginning at 48 hpi (Fig. 5A) and accompanied by differences in UL44 (Fig. 5B) and UL99 (Fig. 5C) levels. These cell-type-dependent differences in RNA levels are consistent with differences detected in viral DNA levels between fibroblasts and epithelial cells. We further evaluated the impact of nitric oxide on viral protein steady-state expression levels. HCMV IE1 (UL123 gene) levels are similar between DETA/NO and control in both cell types (Fig. 5D). However, we observed substantial reductions in pUL44 (UL44 gene) and pp28 (UL99 gene) protein levels beginning at 48 hpi in both cell types. Our data demonstrate that nitric oxide inhibits both viral RNA and protein expression, paralleling drops in genome levels with greater differences detected in epithelial cells than in fibroblasts.

### Nitric oxide reduces oxygen consumption in uninfected and HCMV-infected fibroblasts and epithelial cells.

**FIG 6:**
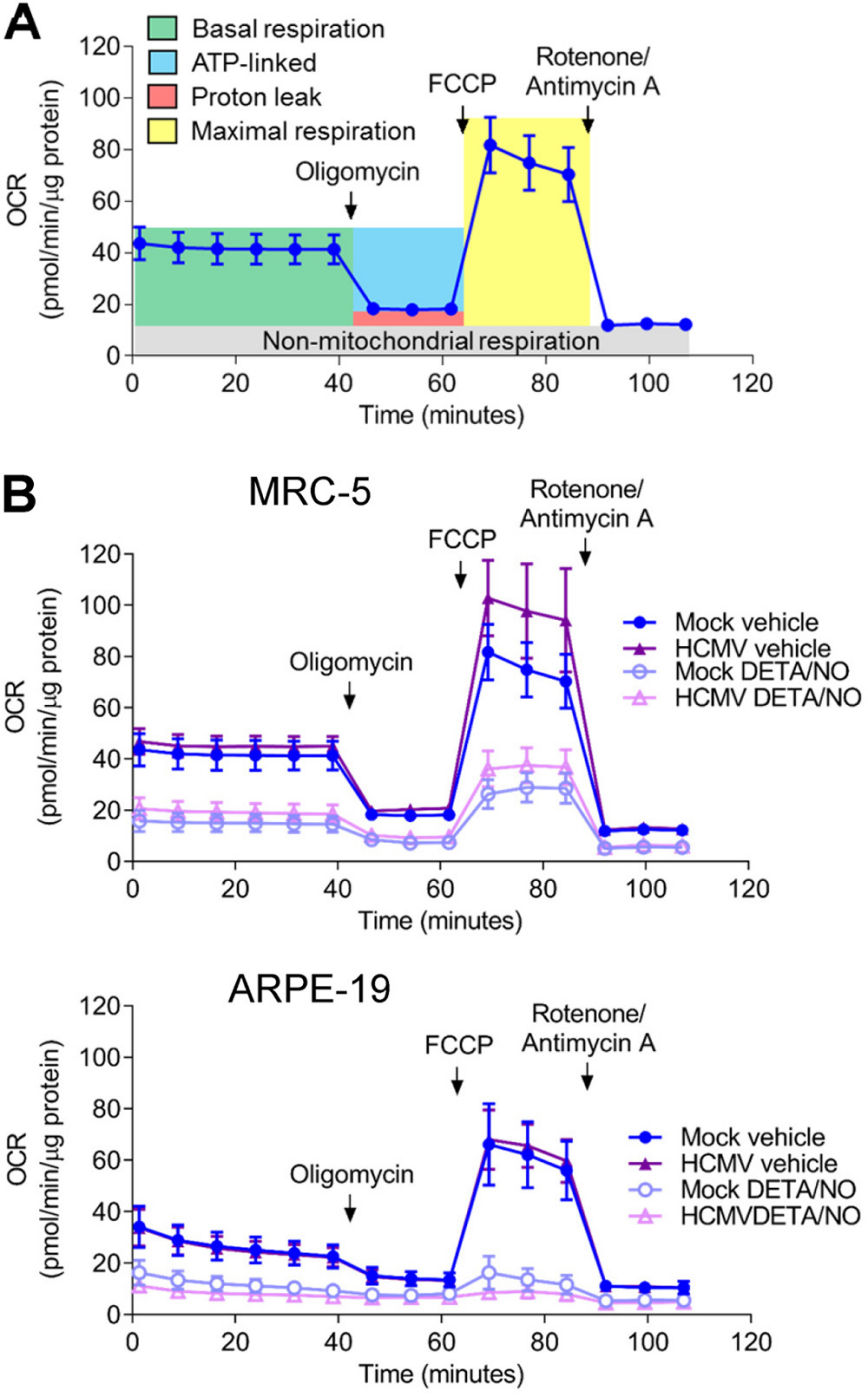
Nitric oxide reduces mitochondrial function. (A) Schematic of mitochondrial stress analysis using extracellular flux technology to measure cellular oxygen consumption. Sequential injections of oligomycin, FCCP, and rotenone/antimycin A allow the measurement of basal (green), ATP-linked (blue), maximal (yellow), proton leak (red), and nonmitochondrial (gray) oxygen consumption rates. (B) Confluent MRC-5 or ARPE-19 cells in a 96-well Seahorse Bioscience dish were infected at an MOI of 3 or 5 U/cell, respectively, or mock infected. At 2 hpi, cells were treated with 500 μM DETA/NO or vehicle control. Mitochondrial stress analysis was performed at 24 hpi as described above and normalized to total micrograms of protein.

**FIG 7:**
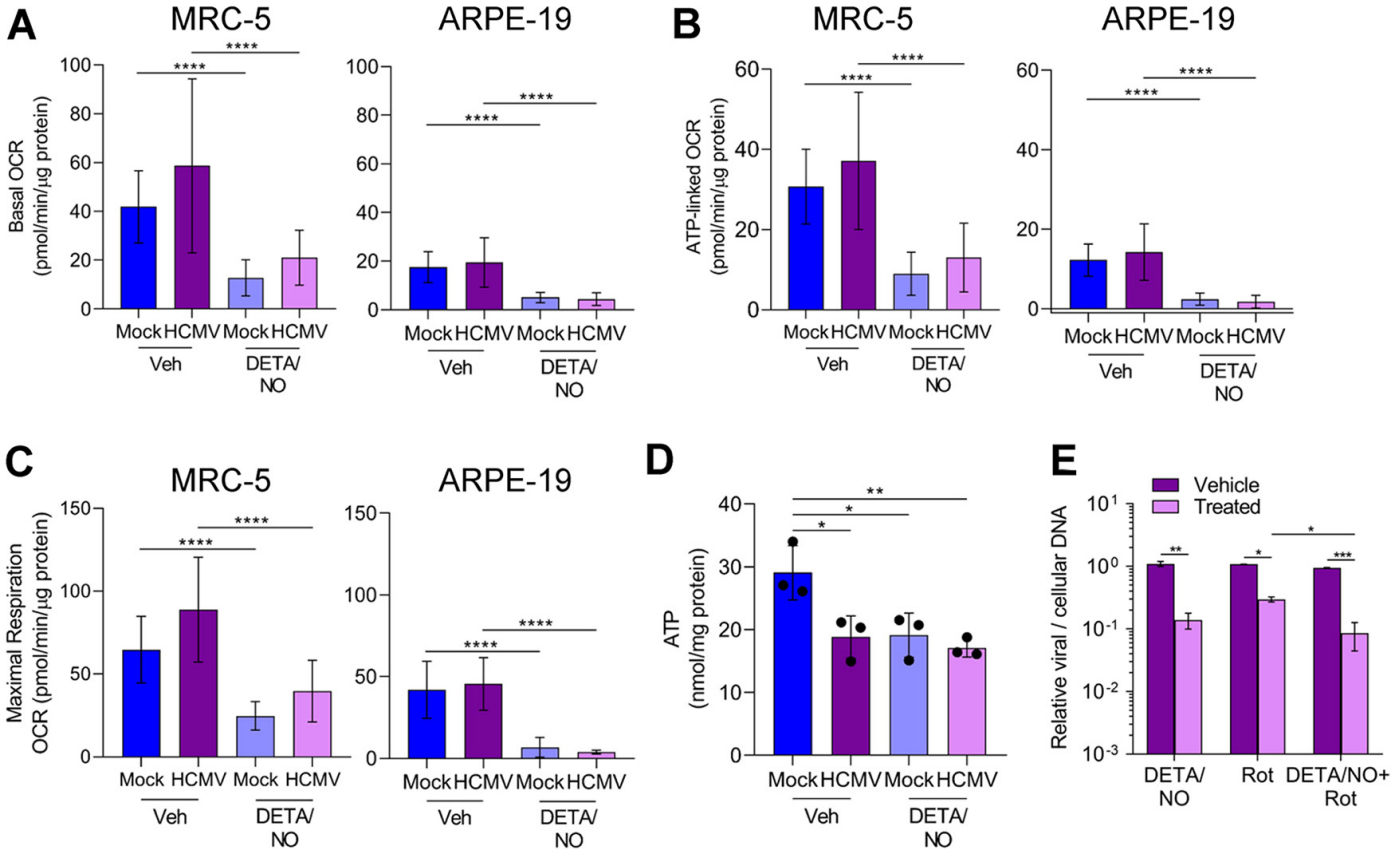
Basal, ATP-linked, and maximal oxygen consumption is reduced during nitric oxide exposure. (A to C) Basal (A), ATP-linked (B), and maximal (C) cellular oxygen consumption was measured for MRC-5 and ARPE-19 cells during mitochondrial stress analysis as described in the legend to Fig. 6. Data are the results of two (ARPE-19) or three (MRC-5) biological replicates and 7 to 8 technical replicates. *P* < 0.0001, ****. (D) MRC-5 cells were plated at subconfluence, serum starved, and infected an MOI of 3 IU/cell or mock infected. Cells were treated at 2 hpi with 500 μM DETA/NO, and ATP was measured at 24 hpi using HPLC. Data are the results of three biological replicates. *P < *0.03, *; *P* = 0.01, **. (E) MRC-5 cells were plated at subconfluence, serum starved for 24 h, and infected with HCMV strain TB40/E-GFP at an MOI of 3 IU/cell. Cells were treated at 2 hpi with 500 μM DETA/NO, 1 μM rotenone, or vehicle control. Relative viral to cellular DNA levels were determined at 24 hpi using primers to HCMV UL123 and cellular TP53 genes. Data represent two biological and two technical replicates. *P* < 0.03, *; *P* = 0.002, **; *P* = 0.0008, ***. Error bars represent standard deviation from the mean. Statistical analyses were performed on transformed data for panels A to C and E. One-way ANOVA (A to D) or two-way ANOVA (E) was used to determine significance. Black circles represent individual data points.

Efficient HCMV replication is dependent on cellular metabolism and mitochondrial respiration (48, 49). Infection induces mitochondrial morphology changes and increases mitochondrial respiration (48, 50). Nitric oxide disrupts the electron transport chain by inhibiting complex I and complex IV (33–35). To determine if nitric oxide disrupts respiration during infection, mitochondrial function was examined using an extracellular flux assay (Fig. 6). Fibroblasts and epithelial cells were plated confluently, infected at an MOI of 3 or 5 IU/cell, respectively, and treated at 2 hpi with 500 μM DETA/NO. At 24 hpi, treated medium was replaced with medium containing only high glucose, and a mitochondrial stress assay was performed. Briefly, oxygen consumption rate (OCR) is measured during sequential injections of oligomycin (ATP synthase inhibitor), carbonyl cyanide 4-(trifluoromethoxy)phenylhydrazone (FCCP; mitochondrion uncoupler), and rotenone/antimycin A (complex I and III inhibitors). This measurement allows the quantitation (Fig. 6A) of basal (green), ATP-linked (blue), maximal (yellow), proton leak (red), and nonmitochondrial (gray) OCRs. Representative plots of the mitochondrial stress assay in fibroblasts and epithelial cells at 24 hpi are shown (Fig. 6B) with the quantified OCR for each measurement (Fig. 7A to C). We observed no significant differences in basal (Fig. 7A), ATP-linked (Fig. 7B), or maximal (Fig. 7C) respiration OCR between uninfected and HCMV-infected cells at 24 hpi. In fibroblasts, basal OCR for uninfected vehicle-treated cells was 41.5 pmol/min/μg protein (Fig. 7A) Upon DETA/NO addition to uninfected cells, we observed significant reductions in basal, ATP-linked, and maximal OCR at 69%, 71%, and 62%, respectively (Fig. 7A to C). Similarly, in HCMV-infected cells, nitric oxide resulted in significant reductions in basal, ATP-linked, and maximal OCR by 64%, 65%, and 55%, respectively (Fig. 7A to C). In uninfected epithelial cells, OCR was substantially reduced from fibroblasts with a basal OCR of 17.5 pmol/min/μg protein (Fig. 7A). Exposure of uninfected epithelial cells to nitric oxide had a larger effect with reductions in basal OCR by 80%, ATP-linked OCR by 71%, and maximal OCR by 84% (Fig. 7A to C). During infection, DETA/NO treatment again reduced basal, ATP-linked, and maximal OCR by 88%, 77%, and 91%, respectively, compared to vehicle treatment (Fig. 7A to C). These data demonstrate that nitric oxide exposure results in reduced mitochondrial function and oxygen consumption rates (OCRs), including ATP-linked OCR, in both uninfected and infected fibroblasts and epithelial cells. These studies also highlight the intrinsic differences between cell types with MRC-5 fibroblasts exhibiting 2.4-fold-higher basal OCR than ARPE-19 epithelial cells.

Reduced mitochondrial function can result in decreased ATP levels if glycolysis is unable to compensate for mitochondrial ATP production. To determine if nitric oxide reduces steady-state ATP levels, we measured intracellular ATP from fibroblasts using high-performance liquid chromatography (HPLC). Subconfluent cells were infected at an MOI of 3 IU/cell or mock infected. We treated samples with 500 μM DETA/NO or vehicle at 2 hpi and measured ATP levels at 24 hpi. In uninfected cells, we measured a 34% reduction in steady-state ATP levels compared to vehicle (Fig. 7D), and this reduction was similar to that observed in untreated, HCMV-infected cells. Upon DETA/NO treatment of HCMV-infected cells, ATP levels were unchanged compared to vehicle at 24 hpi. These data suggest that ATP levels are maintained by alternative pathways during infection and likely not a major contributor to decreased viral DNA levels following nitric oxide exposure.

Because of the pleiotropic effect of nitric oxide, we hypothesized that inhibition of mitochondrial function was not fully responsible for decreased HCMV replication. To determine if other cellular processes are involved, we measured the impact of rotenone, a complex I inhibitor, on HCMV DNA levels at 24 hpi in the presence and absence of nitric oxide exposure. The addition of 500 μM DETA/NO at 2 hpi resulted in a 0.9-log reduction in viral DNA levels at 24 hpi (Fig. 7E) as observed earlier. The addition of 1 μM rotenone resulted in a 0.56-log reduction. When rotenone was combined with nitric oxide exposure, we quantified a significant reduction in viral DNA levels compared to rotenone alone. Taken together, these data demonstrate that nitric oxide disrupts normal mitochondrial function likely through inhibition of the ETC (direct) and/or electron supply to the ETC (indirect) regardless of HCMV infection and that inhibition of mitochondrial function is only partially responsible for decreased viral genome synthesis.

### Metabolite pools are altered in response to nitric oxide exposure during infection.

**FIG 8:**
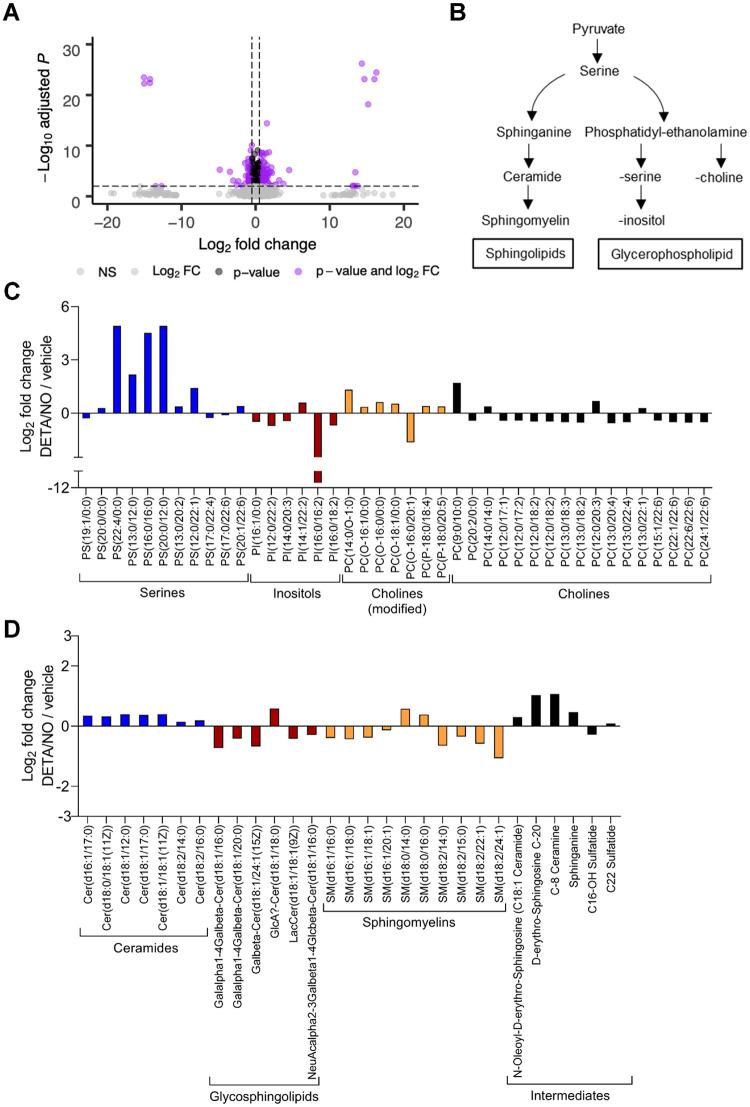
Lipid metabolites relevant to virion production are altered in response to nitric oxide exposure during HCMV infection. MRC-5 cells were plated at subconfluence, serum starved for 24 h, infected at an MOI of 3 IU/cell, and treated at 2 hpi with 500 μM DETA/NO. Cells were washed, collected at 24 hpi into liquid nitrogen, and analyzed using untargeted LC-MS to measure metabolites. (A) Volcano plot of altered metabolites during infection in DETA/NO- versus vehicle-treated groups. Each circle represents one metabolite. Purple indicates significantly (*P < *0.05) altered metabolites with fold change greater than 1.5. (B) An abridged schematic of lipid pathways relevant to observed changes. (C and D) Significantly altered levels of sphingolipids (C) and glycerophospholipids (D) after nitric oxide exposure compared to vehicle during infection. Data are from three biological replicates and three technical replicate injections with significance determined using unpaired *t* test (*P* < 0.05).

In addition to inhibiting the ETC, nitric oxide can modulate various metabolic pathways by altering enzymatic activities. HCMV infection is dependent on modulating cellular metabolism to support replication (5). To determine the impact of nitric oxide on HCMV-dependent changes to host metabolism, we performed an untargeted metabolomics analysis. These studies identified changes in steady-state levels of small molecules within HCMV-infected cells exposed to nitric oxide compared to vehicle-treated infected cells (Fig. 8A). In contrast to targeted metabolomics, untargeted metabolomics identifies putative small molecules using the METLIN database and based on MS1 *m/z* values and retention times (51, 52). Fibroblasts were infected at an MOI of 3 IU/cell and treated with 500 μM DETA/NO at 2 hpi. At 24 hpi, cells were washed, collected into liquid nitrogen, and analyzed using LC-MS. We detected 4,243 small molecules in three biological replicate experiments including multiple technical replicates, including 2,531 molecules with putative identifications (see Table S1 in the supplemental material). We observed 366 significantly (*P < *0.05) altered metabolites upon exposure to nitric oxide with 160 compounds decreased and 206 compounds increased. Metabolic pathways were defined using the KEGG compound database (Fig. S1). Many small-molecule levels were altered but did not reach statistical significance.

10.1128/mBio.02630-20.1 FIG S1 Altered metabolites and associated pathways in HCMV-infected cells during nitric oxide exposure. Schematic of increased (yellow arrow) and decreased (blue arrow) metabolic intermediates and small molecules based in HCMV-infected cells during nitric oxide exposure and metabolic pathways defined using the KEGG Compound Database. Dotted lines indicate indirect relationships, and open arrows indicates carbon sources. Download FIG S1, TIF file, 0.4 MB. Copyright © 2020 Mokry et al. 2020 Mokry et al. This content is distributed under the terms of the Creative Commons Attribution 4.0 International license.

10.1128/mBio.02630-20.3 TABLE S1 Untargeted metabolomics analysis of DETA/NO- to vehicle-treated HCMV-infected MRC-5 cells at 24 hpi. Complete data set of small molecules obtained from three biological replicates and multiple technical injections using untargeted metabolomics including accession numbers, fold changes, retention times, and MS1 composite spectra. Download Table S1, XLSX file, 0.8 MB. Copyright © 2020 Mokry et al. 2020 Mokry et al. This content is distributed under the terms of the Creative Commons Attribution 4.0 International license.

Exposure of HCMV-infected cells to nitric oxide resulted in significant changes in putative lipid metabolites. Infection upregulates lipid synthesis and production of long-chain fatty acids which influences the virion envelope lipid composition and infectivity (5, 53, 54). We observed significant changes in the steady-state levels of sphingolipids and glycerophospholipids (Fig. 8B) following exposure of HCMV-infected cells to DETA/NO (Fig. 8C and D). Liu et al. (54) have demonstrated that uninfected and infected cells at 96 hpi contain similar levels of glycerophospholipids and yet HCMV virions are enriched in phosphatidylethanolamine (PE) and phosphatidylcholine (PC) and reduced in phosphatidylserine (PS) and phosphatidylinositol (PI) (Fig. 8B). Upon treatment, we observed significant increases in PS (Fig. 8C, blue) with reductions in both PC and PI (Fig. 8C, red and black) and no significant change in PE. Sphingolipids are derived from serine and palmityl-CoA involving sphinganine and ceramide intermediates (Fig. 8B). We detected increased levels of ceramides (Fig. 8D, blue) with various fatty acid chain lengths but decreased levels of sphingomyelin lipids (Fig. 8D, orange). High levels of nitric oxide have been previously demonstrated to increase ceramide concentrations, disrupting sphingolipid synthesis and influencing cell proliferation and survival (55; reviewed in reference 56). These data demonstrate that nitric oxide disrupts fatty acid synthesis with significant increases in ceramides and the glycerophospholipid phosphatidylserine.

**FIG 9:**
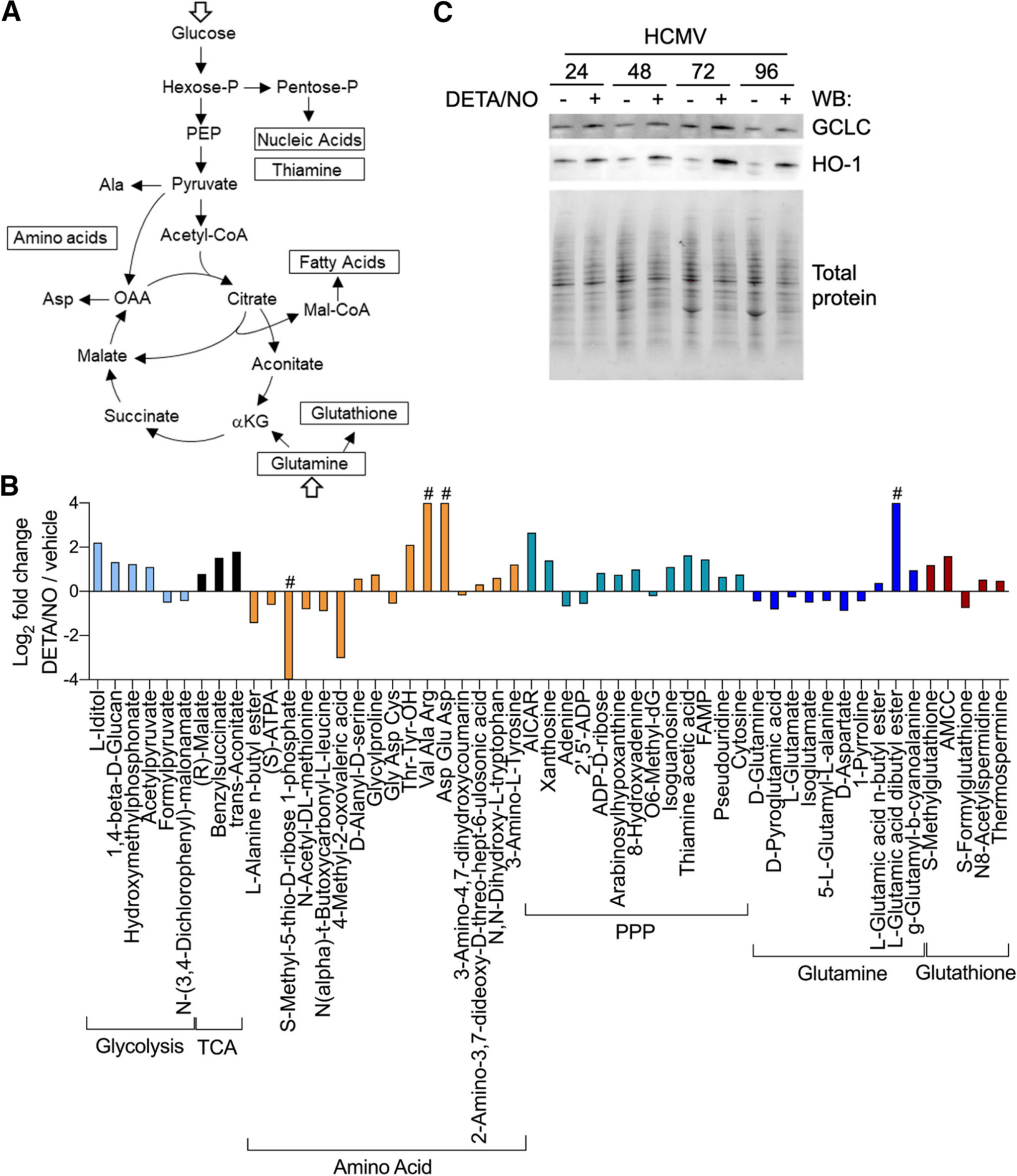
Metabolites in glutamine, TCA cycle, and nucleotide biosynthesis pathways are altered in response to nitric oxide exposure during HCMV infection. (A) Schematic of metabolic pathways during infection (5). (B) As described in the legend to Fig. 8, MRC-5 cells were plated at subconfluence, serum starved for 24 h, infected at an MOI of 3 IU/cell, and treated at 2 hpi with 500 μM DETA/NO. Cells were washed, collected at 24 hpi into liquid nitrogen, and analyzed using untargeted LC-MS to measure metabolites. The graph displays relative abundance of significantly altered metabolites from a subset of metabolic pathways during nitric oxide exposure: glycolysis (light blue), TCA cycle (black), amino acid (orange), pentose phosphate pathway (PPP) (blue/green), glutamine (dark blue), and glutathione (red). Data are from three biological replicates and three technical replicate injections with significance determined using unpaired *t* test (*P* < 0.05). # indicates that the metabolite was identified in only one treatment group. (C) MRC-5 cells were infected and treated as described above. Whole-cell lysates were collected at the indicated time points (hours postinfection, above blot). Lysates were analyzed by Western blotting using antibodies against the indicated cellular proteins. Total protein was used as a loading control. Data represent three biological replicates.

HCMV infection induces glucose transport, consumption, and glycolytic efflux during infection (Fig. 9A) (4–8, 10, 57). We observed several metabolites associated with glycolysis altered during nitric oxide exposure, including increases in l-iditol, hydroxymethyl phosphonate, and acetyl pyruvate by 4.6-, 2.4-, and 2.2-fold, respectively (Fig. 9B, light blue). These molecules are by-products of metabolites in the main glycolytic pathway, suggesting a disruption to glycolysis. Infection also activates the TCA cycle (5), and we observed increases in three compounds related to the TCA cycle: *trans*-aconitate, benzylsuccinate, and (*R*)-malate, which increased by 3.5-, 2.9-, and 1.7-fold, respectively (Fig. 9B, black). Considering that the enzyme aconitase is a known target of nitric oxide (34), these steady-state increases suggest a disruption in the TCA cycle at the transition between citrate and isocitrate.

We also identified changes in both nucleotide and amino acid metabolism. Several putative amino acid metabolites were altered including di- and tripeptides, suggesting any HCMV-induced reprogramming of amino acid metabolism is dysregulated upon nitric oxide exposure (Fig. 9B, orange). HCMV also upregulates nucleotide biosynthesis (58). We observed that treatment with nitric oxide resulted in an overall increase in nucleotide metabolites including AICAR (5-amino-4-imidazolecarboxamide ribose) by 6.3-fold, cytosine by 1.7-fold, and pseudouridine by 1.6-fold (Fig. 9B, blue/green). Derivatives of guanine increased, including xanthosine, isoguanine, and hypoxanthine arabinoside by 2.6-, 2.2-, and 1.7-fold, respectively. Importantly, isoguanine and hypoxanthine arabinoside have been shown to inhibit herpes simplex virus replication while AICAR can disrupt HCMV replication (59–61). Adenine was observed to decrease by 0.6-fold, and its derivatives 8-hydroxyadenine and ADP-d-ribose increased by 2.0- and 1.8-fold, respectively. These data demonstrate that nitric oxide dysregulates amino acid and nucleotide metabolism, including significantly increased levels of nucleotides previously shown to inhibit replication of herpesviruses.

Glutamine uptake and glutaminolysis are induced during HCMV infection, and glutamine deprivation results in a loss of virus production (5, 9, 10). It has been proposed that glutaminolysis is required to replenish TCA cycle pools during infection through conversion of glutamine to α-ketoglutarate (Fig. 9A) (5, 11, 12). We observed that nitric oxide exposure decreases putative glutamine-related metabolites during infection (Fig. 9B, dark blue). Glutamine and glutamate were significantly decreased by 0.7- and 0.8-fold, respectively. Additionally, aspartate was reduced by 0.5-fold and other intermediates in glutamine metabolism were reduced, including pyroglutamic acid by 0.6-fold, 5-l-glutamyl-l-alanine by 0.7-fold, and isoglutamate by 0.7-fold. These data suggest glutaminolysis is disrupted during nitric oxide exposure with glutamine metabolites being reduced. Glutamine contributes to numerous metabolic pathways including the synthesis of glutathione, which is an antioxidant molecule stimulated by nitric oxide and aids in restoration of mitochondrial function (62). Glutathione has been demonstrated to increase during HCMV infection (10). We observed changes in several putative metabolites involved in glutathione metabolism upon exposure to nitric oxide. (Fig. 9B, red). These molecules include S-methylglutathione by 2.3-fold, AMCC [N-acetyl-S-(N-methylcarbamoyl)-l-cysteine] by 3.0-fold, S-formylglutathione by 0.6-fold, and thermospermine by 1.4-fold. The increase in glutathione metabolites is consistent with nitric oxide exposure and may contribute to the steady-state decrease in glutamine, a known precursor of glutathione.

We next asked whether enzymes required for glutathione production were increased during nitric oxide exposure in HCMV-infected cells. Activation of the redox transcription factor NRF2 and its downstream target glutamate-cysteine ligase catalytic subunit (GCLC) is required for glutathione synthesis from its precursor glutamine, while heme oxygenase 1 (HO-1) is indicative of NRF2 activation. We observed increased levels of HO-1 and GCLC beginning at 24 hpi in HCMV-infected cells during nitric oxide exposure (Fig. 9C). These data are in agreement with increased glutathione metabolites accompanied by a decrease in the glutathione precursor glutamine. Cumulatively, our untargeted metabolomics analysis reveals nitric oxide has a wide range of effects on infected cellular metabolism including altered lipid metabolism, increased aconitate, and metabolites associated with nucleotide synthesis (Fig. S1). In addition, nitric oxide exposure increases enzymes required for glutathione production, which coincides with increased glutathione metabolites and decreased glutamine/glutamine intermediates. These data suggest a shuttling of glutamine away from supplying TCA cycle intermediates during infection to the production of the antioxidant glutathione.

### Glutamine deprivation during HCMV infection mimics the effects of nitric oxide.

**FIG 10:**
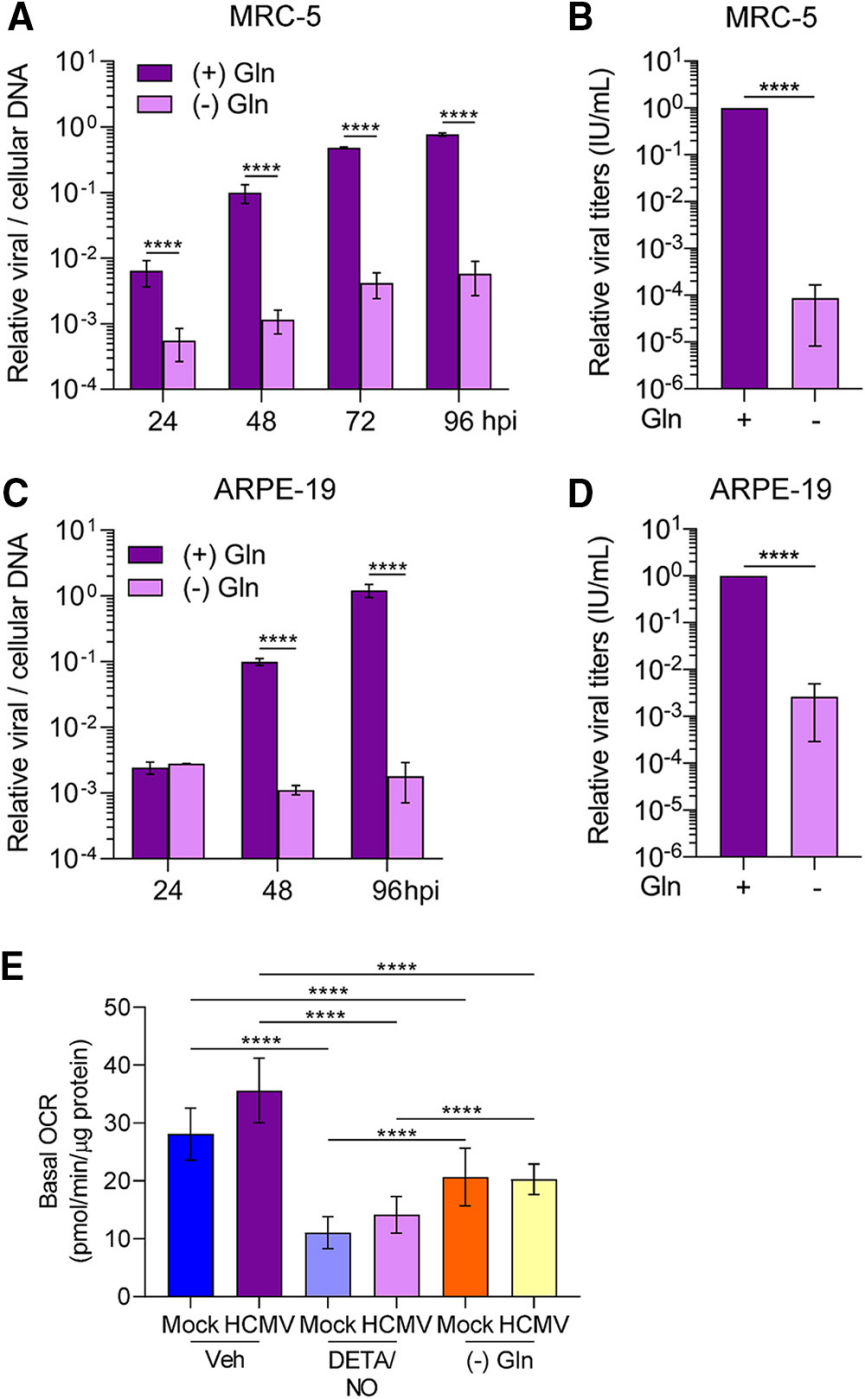
Nitric oxide inhibition of HCMV infection is phenotypically similar to glutamine deprivation. (A to D) MRC-5 cells were plated at subconfluence, serum starved for 24 h, and infected at an MOI of 3 IU/cell (A and B). Confluent ARPE-19 cells were infected at an MOI of 5 IU/cell (C and D). At 2 hpi, cells were washed, and medium was replaced with high glucose with or without 2 mM glutamine. Medium was changed every 24 h to match nitric oxide exposure conditions. Relative viral to cellular DNA levels were determined using quantitative PCR at the indicated time points using primers to HCMV UL123 and cellular TP53 genes (A and C). Viral titers were quantified at 96 hpi by a TCID_50_ assay (B and D). Data are the results of three biological replicates and two technical replicates. (E) MRC-5 cells were plated in a 96-well Seahorse Bioscience dish, grown to confluence, and infected at an MOI of 3 IU/cell or mock infected. At 2 hpi, cells were treated in medium containing high glucose and 2 mM glutamine with 500 μM DETA/NO or vehicle control or with high glucose in glutamine-free medium. Basal oxygen consumption rates were measured at 24 hpi and normalized to total micrograms of protein. Data are the results of two biological replicates and 7 to 8 technical replicates. Data from one replicate were previously displayed in Fig. 7A. Error bars represent standard deviation from the mean. Statistical analyses were performed on transformed data for panels A and C. Two-way ANOVA (A and C), paired *t* test (B and D), or one-way ANOVA (E) was used to determine significance. *P* < 0.0001, ****.

HCMV replication is highly dependent on glutamine (9), and our data demonstrate that nitric oxide exposure decreases glutamine/glutamine metabolites possibly by diverting them to other pathways. To indirectly investigate this possibility, we compared the impact of nitric oxide to that of glutamine deprivation on HCMV replication. We examined viral DNA levels and infectious virus production during glutamine deprivation. Fibroblasts and epithelial cells were infected at an MOI of 3 or 5 IU/cell, respectively. At 2 hpi, cells were washed, and medium was replaced using glucose-containing medium with or without 2 mM glutamine. DNA levels were determined at 24 to 96 hpi, and viral titers were quantified at 96 hpi. In fibroblasts without glutamine, we observed reduced viral DNA levels at all time points including 24 hpi with the largest difference at 96 hpi being a 2.1-log reduction (Fig. 10A). This decrease was accompanied by a 4.2-log reduction in viral titers (Fig. 10B) with a mean of 1.243 × 10^6^ IU/ml with glutamine and 74 IU/ml during glutamine deprivation. In epithelial cells, we observed significant reduction of viral DNA starting at 48 hpi with a 2.9-log reduction by 96 hpi (Fig. 10C). Further, infectious virus production was reduced by 2.8 logs (Fig. 10D) with a mean of 6.3867 × 10^4^ IU/ml with glutamine and 1.013 × 10^2^ IU/ml during glutamine deprivation. Similar to cell-type-dependent differences observed during nitric oxide exposure, these data demonstrate that removal of glutamine from epithelial cells results in a larger reduction in viral DNA levels compared to fibroblasts. However, a larger reduction of infectious virus was observed in glutamine-deprived fibroblasts than in epithelial cells.

We next asked whether glutamine deprivation would also impact mitochondrial respiration. As completed for nitric oxide, we measured OCR during glutamine deprivation (Fig. 10E). Fibroblasts were infected with an MOI of 3 IU/cell or mock infected and treated using either vehicle control with glutamine or 500 μM DETA/NO with glutamine, or untreated without glutamine. We observed a decrease in basal OCR in both uninfected and HCMV-infected glutamine-deprived cells compared to cells supplemented with glutamine. However, the OCRs were higher than those observed during DETA/NO treatment. These data demonstrate that glutamine deprivation significantly reduces mitochondrial respiration but not to the levels observed during nitric oxide exposure.

**FIG 11:**
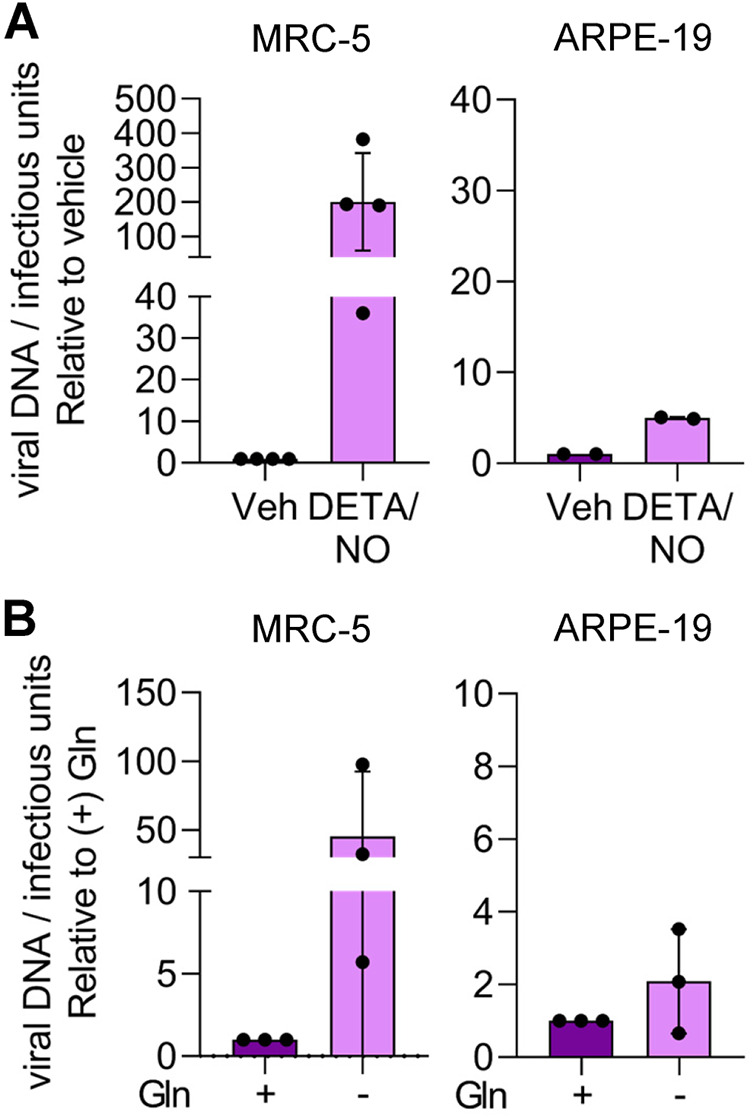
Cell-type-dependent differences in infectivity during nitric oxide exposure and glutamine deprivation. MRC-5 cells were plated at subconfluence, serum starved for 24 h, and infected at an MOI of 3 IU/cell. Confluent ARPE-19 cells were infected at an MOI of 5 IU/cell. At 2 hpi, cells were treated with 500 μM DETA/NO or vehicle control every 24 h (A), or medium was replaced with high glucose with or without 2 mM glutamine (B). Medium was changed every 24 h. Virus infectivity was measured by quantifying relative viral DNA levels to infectious units at 96 hpi. Total DNA was isolated from DNase-treated culture supernatants and measured using quantitative PCR, and viral titers were quantified by a TCID_50_ assay. Data are graphed relative to vehicle (A) or with addition of glutamine (B).

Finally, we hypothesized that the differences in the resulting viral titers between fibroblasts and epithelial cells during nitric oxide exposure and glutamine deprivation were related to infectivity of the viral particles. To assess infectivity, we determined the ratio of viral DNA to infectious units (IU). Fibroblasts and epithelial cells were infected, and cells were treated at 2 hpi with 500 μM DETA/NO or vehicle control. Alternatively, infected cells were cultured in medium with or without 2 mM glutamine. Viral titers were determined at 96 hpi by 50% tissue culture infective dose (TCID_50_) assay, and total DNA was isolated and quantified from DNase-treated culture supernatants as an indirect measurement of viral particles. In infected fibroblasts treated with nitric oxide, we observed an average 200-fold increase in the ratio of viral genomes to infectious units compared to vehicle control (Fig. 11A). In contrast, we detected a 5-fold difference following treatment of epithelial cells with DETA/NO. Upon glutamine removal, we quantified an average 50-fold increase in the ratio of viral genomes to infectious units from fibroblasts and a 2-fold increase from epithelial cells (Fig. 11B). Although we observed substantial and consistent changes in the ratios from multiple biological replicate experiments, the data were variable. These data demonstrate that nitric oxide exposure results in a greater increase in defective viral particles from fibroblasts than from epithelial cells and are parallel to those obtained following glutamine deprivation. Considering the observations of our metabolomics studies and the observed similarities in viral replication defects, we propose that disruption of glutamine metabolism is a likely component of the mechanism in nitric oxide inhibition of HCMV infection.

### Addition of specific intermediates partially restores viral DNA synthesis and infectious virus production.

**FIG 12:**
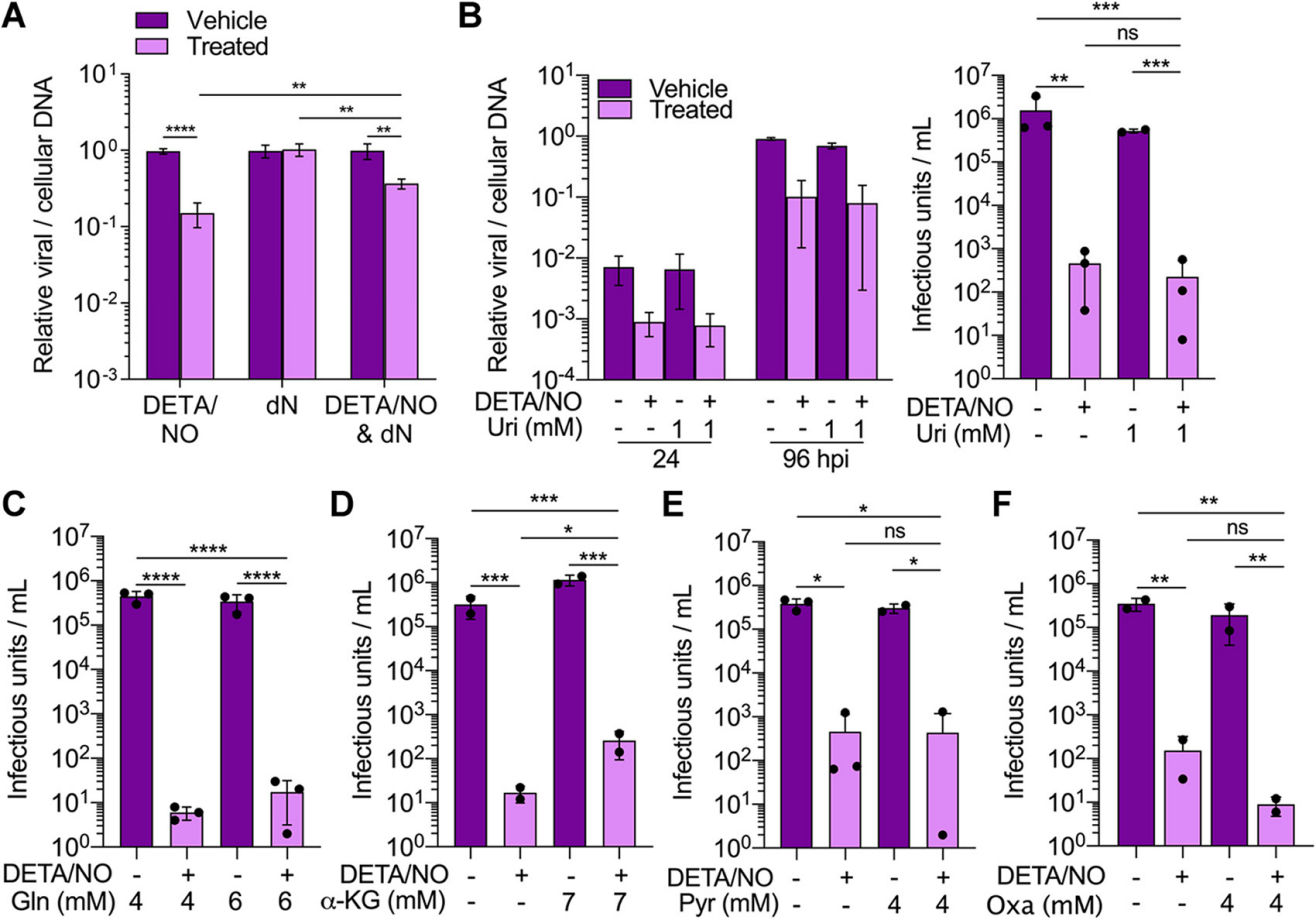
Addition of specific intermediates during nitric oxide exposure partially restores viral DNA synthesis and infectious virion production. (A) MRC-5 cells were plated subconfluently, serum starved for 24 h, and infected at an MOI of 3 IU/cell. At 2 hpi, cells were treated with 500 μM DETA/NO, 1 mM deoxyribonucleosides (dN), or vehicle control. Relative viral to cellular DNA levels were measured at 24 hpi using quantitative PCR with primers to HCMV UL123 and cellular TP53 genes. Data are the results of three biological and two technical replicates. Statistical analyses were performed on transformed data, and two-way ANOVA was used to determine significance. *P* < 0.005, **; *P* < 0.0001, ****. (B to F) Cells were plated subconfluently, serum starved for 24 h, and infected at an MOI of 3 IU/cell. At 2 hpi, cells in high-glucose and 2 mM glutamine medium were treated with 500 μM DETA/NO, or vehicle control, and the addition of 1 mM uridine (B), 2 mM glutamine (C), 7 mM α-ketoglutarate (α-KG) (D), 4 mM pyruvate (E), 4 mM oxaloacetate (Oxa) (F), or vehicle control. (B) Relative viral to cellular DNA levels were measured at 24 and 96 hpi using quantitative PCR with primers to HCMV UL123 and cellular TP53 genes. (B to F) Viral titers were quantified by a TCID_50_ assay using culture supernatants from 96 hpi. Error bars represent standard deviations from the means. Data are the results of two to three biological replicates and two technical replicates. Statistical analyses were performed on transformed data, and one-way ANOVA was used to determine significance. *P* ≤ 0.02, *; *P* ≤ 0.003, **; *P* ≤ 0.0005, ***; *P* < 0.0001, ****. ns, not significant.

Our data demonstrate that nitric oxide has a pleiotropic effect on cellular processes required for HCMV replication including disruptions in mitochondrial function, metabolism, and cell proliferation. Because of these observations, we postulated that we could partially but not fully rescue viral replication by supplementing metabolites within specific pathways. To address disruptions to nucleotide metabolites, we measured changes in viral DNA levels at 24 hpi following the addition of deoxynucleosides. Fibroblasts were infected at an MOI of 3 IU/cell and treated at 2 hpi with 500 μM DETA/NO, vehicle control, and/or medium containing 1 mM deoxynucleosides. We measured a 0.4-log reduction in viral DNA levels when supplementing with deoxynucleosides compared to a 0.8-log reduction with DETA/NO alone (Fig. 12A). These data demonstrate that addition of deoxynucleosides provides some complementation to the defects in viral DNA synthesis caused by nitric oxide exposure. The addition of uridine has been previously demonstrated to rescue HCMV replication upon inhibition of pyrimidine biosynthesis (58). Next, we tested if supplementing with uridine could restore replication. Cells were infected and treated as described above in medium supplemented with 1 mM uridine. Because uridine enters the pentose phosphate pathway and influences virus production (58), we analyzed viral DNA levels at 24 and 96 hpi and virus production. However, unlike deoxynucleosides, the addition of uridine to the culture medium failed to restore HCMV replication (Fig. 12B).

Next, we focused on complementing defects in glutamine-dependent activities by supplementing medium with either additional glutamine, α-ketoglutarate, pyruvate, or oxaloacetate during nitric oxide exposure. Previous studies by Chambers et al. (9) demonstrated that addition of α-ketoglutarate, pyruvate, or oxaloacetate rescued virus production during glutamine deprivation. At 2 hpi, infected fibroblasts were treated with 500 μM DETA/NO or vehicle control in medium containing glucose with 4 mM glutamine or an additional 2 mM glutamine. We measured viral DNA levels and titers at 96 hpi and observed similar reductions in viral DNA levels (Fig. S2A) and viral titers regardless of additional glutamine (Fig. 12C). For the other metabolites, infected fibroblasts were treated at 2 hpi with 500 μM DETA/NO or vehicle control and cultured in medium supplemented with 7 mM α-ketoglutarate, 4 mM pyruvate, or 4 mM oxaloacetate. We again measured viral DNA and titers at 96 hpi. Supplementing with either α-ketoglutarate, pyruvate, or oxaloacetate did not restore viral DNA levels to those measured in vehicle-treated samples (Fig. S2B to D). However, the addition of α-ketoglutarate resulted in a 1-log increase in viral titers during nitric oxide exposure (Fig. 12D), while addition of pyruvate or oxaloacetate did not restore virus production (Fig. 12E and F). These results demonstrate that supplementing medium with α-ketoglutarate partially overcomes nitric oxide-mediated disruption of HCMV replication. Overall, these data show that inhibition of HCMV replication by nitric oxide can be partially rescued by two metabolites, deoxynucleosides and α-ketoglutarate. In addition, these observations further support the hypothesis that viral inhibition by nitric oxide involves disruptions to multiple cellular processes.

10.1128/mBio.02630-20.2 FIG S2 Viral genome synthesis is not restored after addition of intermediates during nitric oxide exposure. MRC-5 cells were plated subconfluently, serum starved for 24 h, and infected at an MOI of 3 IU/cell. At 2 hpi, cells in high-glucose and 4 mM glutamine medium were treated with 500 mM DETA/NO, or vehicle control, and the addition of 2 mM glutamine (Gln) (A), 7 mM α-ketoglutarate (α-KG) (B), 4 mM pyruvate (C), 4 mM oxaloacetate (Oxa) (D), or vehicle control. Relative viral to cellular DNA levels were measured at the indicated time points using quantitative PCR with primers to HCMV UL123 and cellular TP53 genes. Data are the results of two to three biological and two technical replicates. Download FIG S2, TIF file, 0.9 MB. Copyright © 2020 Mokry et al. 2020 Mokry et al. This content is distributed under the terms of the Creative Commons Attribution 4.0 International license.

## DISCUSSION

In addition to its role as a vasodilator, nitric oxide released from innate immune cells contributes to suppressing infections. Its importance against HCMV is highlighted by a recent clinical case describing an adult male with a NOS2 deficiency who died due to an active HCMV infection (23). Prior to our studies, however, the mechanism of nitric oxide inhibition of HCMV replication remained largely unknown. Nitric oxide is a selectively reactive molecule with high reactivity to two types of targets, transition metal ions and other free radicals. Its biological chemistry with other metal sites and free radicals can result in a complex biological chemistry consisting of multifaceted and often contradictory outcomes. We have demonstrated that nitric oxide attenuates HCMV infection by suppressing mitochondrial respiration and altering metabolite pools. Further, nitric oxide exposure results in a cell cycle arrest, suggesting immunopathogenic effects on uninfected cells and likely contributing to the observed defects in HCMV replication.

HCMV replication is highly reliant on glutamine metabolism with infection increasing glutamine uptake and consumption (9). Glutamine deprivation results in inhibition of infectious virus production which can be rescued by the addition of glutamine, pyruvate, oxaloacetate, or α-ketoglutarate to the culture medium (9). Similarly, nitric oxide exposure reduces glutamine and glutamine intermediates (Fig. 9B), attenuates viral DNA synthesis (Fig. 1B and C), and significantly reduces infectious virus production (Fig. 1E). However, unlike in studies on glutamine deprivation, only the addition of α-ketoglutarate exhibited a partial rescue following exposure (Fig. 12D). Decreased glutamine intermediates during nitric oxide exposure could occur in several ways. Studies by Rodríguez-Sánchez et al. (10) demonstrated that a virus deficient in HCMV pUL38 exhibits reduced glutamine consumption. We observed that nitric oxide reduces viral gene and protein expression (Fig. 5), suggesting that suppression of viral gene expression is a likely contributor to the observed metabolic defects. Alternatively, glutamine may be shuttled to other metabolic pathways. Glutamine is a precursor to the antioxidant glutathione which is induced upon activation of the redox transcription factor NRF2. We detected increased levels of glutathione-related metabolites upon nitric oxide treatment (Fig. 9B). In addition, we observed increased steady-state levels of NRF2-regulated antioxidant enzymes, heme oxygenase 1 (HO-1) and glutamate-cysteine ligase catalytic subunit (GCLC), in the presence of nitric oxide compared to vehicle-treated infections (Fig. 9C). HO-1 and GCLC have been observed to increase during a normal infection (63, 64). Finally, glutamine is required for the conversion of aspartate to asparagine by asparagine synthetase (ASNS), which contributes to efficient HCMV replication (65). We detected decreased levels of aspartate upon exposure to nitric oxide, and it is possible that glutamine is being shuttled into asparagine (Fig. 9B). Future studies will evaluate nitric oxide-mediated changes in these specific metabolic pathways. Overall, our studies indicate that nitric oxide disruption of HCMV replication involves altered glutamine metabolism and support the hypothesis that multiple mechanisms are involved.

HCMV replication is also reliant on cellular nucleotides for genome synthesis (5, 7, 58, 66, 67). Nucleotide biosynthesis is dysregulated during nitric oxide exposure including an increase in the DNA damage marker isoguanine (Fig. 9B). Other nucleotides increased during nitric oxide exposure have been previously demonstrated to disrupt DNA synthesis. Arabinosyl hypoxanthine has antiviral effects against several herpesviruses including HCMV (59), and incorporation of 8-hydroxyadenine during DNA synthesis can result in DNA errors (68). In addition, we quantified a significant increase in AICAR, an activator of AMP-activated kinase (AMPK) (Fig. 9B). AICAR is known to disrupt HCMV replication (61). Our deoxynucleoside addback experiment partially restored viral genome synthesis at 24 hpi (Fig. 12A). However, the uridine addback failed to rescue the defects (Fig. 12B). Deoxynucleosides are converted to deoxynucleotides through salvage pathways that do not involve the nitric oxide target, ribonucleotide reductase (RNR) (36, 37). Kuny and Kalejta (66) demonstrated that hydroxyurea-mediated inhibition of RNR disrupts HCMV replication and is rescued by addition of deoxynucleosides. We cannot rule out the involvement of RNR in nitric oxide-mediated inhibition of genome synthesis. Our work indicates that nitric oxide exposure increases unique nucleotide metabolites by 24 hpi with several being known inhibitors of viral replication.

Mitochondrial respiration occurs through the flow of electrons through the ETC that facilitates the movement of protons from the mitochondrial matrix to the intermembrane space and ultimately drives ATP synthesis. Electron carriers, NADH and FADH_2_, from the TCA cycle and other metabolic pathways donate electrons to complex I and complex II of the ETC, respectively. Mitochondrial respiration was severely inhibited in both uninfected and HCMV-infected cells during nitric oxide exposure (Fig. 6 and 7). Mitochondrial biology plays a significant role in supporting HCMV replication. Infection stimulates mitochondrial biogenesis and respiration in a process that is partially dependent on HCMV UL37x1 (48, 50). Consistent with our studies, Combs et al. (50) demonstrated that basal oxygen consumption rates are increased in untreated HCMV-infected cells starting at 48 hpi but not at 24 hpi (Fig. 7). Moreover, disruption of the ETC and respiration through depletion of mitochondrial DNA (mtDNA) inhibits virus production with little effect on viral protein expression (50). In contrast, we observed significant reductions in early and late viral protein levels due to nitric oxide (Fig. 5D). We also demonstrated a significantly greater decrease in viral DNA levels when the complex I inhibitor rotenone is combined with nitric oxide compared to complex I inhibition alone (Fig. 7E). Importantly, complex II is still active under these conditions, suggesting nitric oxide may limit electron donors for the ETC. These data further support the hypothesis that multiple mechanisms are involved in nitric oxide inhibition of HCMV. The contribution of the ETC to HCMV replication is currently unknown but likely involves production of ATP to meet increased energy requirements. We observed that ATP levels in HCMV-infected cells during nitric oxide exposure were unchanged compared to vehicle control (Fig. 7D), indicating compensation from an alternate pathway. The viral noncoding immediate early RNA β2.7 has previously been shown to prevent rotenone-induced ATP depletion (69). It is conceivable that expression of β2.7 RNA or virion-delivered β2.7 (70, 71) is protecting ATP production during nitric oxide exposure. Further, increased glycolysis can compensate for decreased mitochondrial function, and our metabolomic data indicate several glycolytic intermediates are increased during nitric oxide exposure (Fig. 9B). Future studies will elucidate the activities of HCMV β2.7 and glycolysis during nitric oxide-mediated inhibition of HCMV.

Nitric oxide decreases HCMV genome synthesis, gene expression, and infectious virus production in both fibroblasts and epithelial cells (Fig. 1 and 3). However, we observed differences between cell types. Nitric oxide had substantially reduced viral genome levels in epithelial cells compared to fibroblasts. Yet production of infectious virus was dramatically reduced in fibroblasts compared to epithelial cells. Our data indicate that this difference in titers is the result of more defective particles produced from the treated fibroblasts (Fig. 11A). These same trends were observed in our glutamine-deprivation studies (Fig. 10 and 11B). At 96 hpi, virus production was ∼10^5^ to 10^6^ IU/ml in fibroblasts and ∼10^3^ to 10^4^ IU/ml in epithelial cells. These data show differences in infectivity between the cell types which may contribute to variation in the effect of nitric oxide exposure. Another possible contributor to these observations is differences between host cell metabolism including lipid metabolism. Liu et al. (54) have defined the lipid composition of the viral particle and postulate that fatty acids and lipids synthesized during infection are the major contributor to the lipid composition of the viral particle (54). HCMV does remodel the host lipidome, which is partially mediated by expression of HCMV UL37x1 (72). We detected changes in host cell lipids at 24 hpi between vehicle- and nitric oxide-treated fibroblasts (Fig. 8). Intrinsic differences in metabolic regulation are evidenced by the 2.4-fold difference in basal OCR between fibroblasts and epithelial cells (Fig. 7A). It is conceivable that HCMV modulation of host cell metabolism differs between cell types, impacting the susceptibility of infected cells to the antiviral activities of nitric oxide. It is unknown how nitric oxide disruption of HCMV-mediated metabolic regulation will affect cell types that are more sensitive to nitric oxide such as endothelial and smooth muscle cells. Further, while nitric oxide is critical for the establishment and maintenance of viral latency in hematopoietic progenitor cells (30), the contribution of disrupted metabolism is not known. Additional studies will elucidate these mechanisms.

Nitric oxide is an important regulator of chronic infection and is likely produced at high levels during HCMV infection and reactivation. In addition to its antiviral role, our data indicate an immunopathogenic role of nitric oxide, having shown both cytostasis and decreased mitochondrial respiration in uninfected fibroblasts and epithelial cells (Fig. 4, 6, and 7). Because of observations similar to ours, nitric oxide is often referred to as a “double-edge sword.” The inflammatory role of nitric oxide has been observed in numerous disease models including irritable bowel syndrome and neurodegenerative diseases (73, 74), emphasizing the potential immunopathogenic effect of nitric oxide despite its protective role during viral infection. We have demonstrated that nitric oxide inhibition of HCMV is multifactorial and involves inhibition of mitochondrial respiration and dysregulation of HCMV metabolic control.

## MATERIALS AND METHODS

### Cell culture and virus.

MRC-5 fibroblasts and ARPE-19 epithelial cells (ATCC) were cultured in Dulbecco’s modified Eagle medium (DMEM) (ThermoFisher) containing 7% fetal bovine serum (FBS) (Atlanta Biologicals) and 1% penicillin-streptomycin (P/S) (ThermoFisher) at 37°C under 5% CO_2_. Cells were plated at approximately 50 to 60% confluence and serum starved using 0.5% FBS for 24 h to synchronize the cell cycle prior to infection.

Viral stocks of HCMV strain TB40/E encoding GFP were produced by transfecting a bacterial artificial chromosome (BAC) encoding the virus and a plasmid encoding UL82 into MRC-5 fibroblasts using electroporation at 260 mV for 30 ms with a 4-mm-gap cuvette and a Gene Pulser XCell electroporation system (75–78). Virus was propagated in fibroblasts, and medium was collected and pelleted through a sorbitol (20% sorbitol, 50 mM Tris-HCl, pH 7.2, 1 mM MgCl_2_) cushion at 55,000 × *g* for 1 h in a Sorvall WX-90 ultracentrifuge and SureSpin 630 rotor (Thermo Fisher Scientific). Viral stock titers were quantified on MRC-5 cells in 96-well dishes using a limiting dilution assay (TCID_50_). HCMV IE1-positive cells were determined at 2 weeks postinfection, and resulting titers were referred to as infectious units per milliliter (IU/ml). An MOI of 3 or 5 was used for infection of MRC-5 and ARPE-19 cells, respectively. Cells were washed 2 hpi with phosphate saline buffer (PBS) (Thermo Fisher Scientific), and new medium was added.

### Chemical reagents and antibodies.

Diethylenetriamine NONOate (DETA/NO) (Cayman Chemicals) was maintained in 0.01 M NaOH at a concentration of 50 mM at −20°C. Spent DETA/NO was prepared by diluting a 50 mM stock of DETA/NO to 500 μM in high-glucose, 2 mM glutamine DMEM with 7% FBS, and 1% P/S and incubating at 37°C for 48 h. Spent DETA/NO was then maintained at 4°C. Deoxynucleosides 2′-deoxyadenosine monohydrate, thymidine, and 2′-deoxycytidine hydrochloride (Millipore Sigma) were prepared fresh just prior to treatment and dissolved in high-glucose, 4 mM glutamine DMEM (Thermo Fisher Scientific) containing 7% FBS, and 1% P/S, while 2′-deoxyguanosine monohydrate (Millipore Sigma) was dissolved in 0.01 M NaOH. Medium was filter sterilized after addition of deoxynucleosides. Glutamine (ThermoFisher), α-ketoglutaric acid (Millipore Sigma), sodium pyruvate (ThermoFisher), oxaloacetic acid (Millipore Sigma), and uridine (Millipore Sigma) were maintained at 4°C in high-glucose, 4 mM glutamine DMEM containing 7% FBS, and 1% P/S at 2, 7, 4, 4, and 1 mM, respectively. Rotenone (a generous gift from John Corbett, The Medical College of Wisconsin) was maintained in dimethyl sulfoxide (DMSO) at a concentration of 5 mM at −20°C. cPTIO was maintained in H_2_O at a concentration of 50 mM at −20°C. To assess compound toxicity, cells were plated at approximately 50 to 60% confluence and treated with a range of DETA/NO (Cayman Chemicals) concentrations or 0.01 M NaOH as vehicle control or not treated. Treatments were replaced every 24 h. Samples were collected at 24, 48, 72, and 96 h posttreatment, and cytotoxicity was assessed using trypan blue exclusion with live cell count and cell viability being reported. A Countess automated cell counter (Invitrogen) and hemocytometer were used for cell counts.

For Western blot analysis (WB), antibodies and dilutions used were the following: mouse anti-pUL44 (clone 10D8, 1:2,500; Virusys), rabbit anti-cyclin E1 (clone HE12, 1:1,000; CST), rabbit anti-cyclin B1 (clone D5C10, 1:1,000; CST), mouse anti-p21 (clones CP36 and CP74, 1:1,000; Millipore Sigma), mouse anti-GCLC (clone OTI1A3, 1:300; Thermo Fisher Scientific), and rabbit anti-HO-1 (1:1,000; Enzo Life Sciences). The HCMV antibodies mouse anti-pUL123 (clone 1B12, 1:1,000) and mouse anti-pp28 (clone 10B4, 1:1,000) were generously provided by Tom Shenk (Princeton University). Goat anti-mouse and goat anti-rabbit immunoglobulin (IgG) conjugated to horseradish peroxidase (HRP) (Jackson Immunoresearch) were used for secondary antibody at 1:10,000 dilution.

### Analysis of protein and nucleic acid.

Quantitative PCR (qPCR) was used to determine cellular and viral DNA and RNA contents. For DNA studies, cells were washed with PBS, collected by trypsinization, and isolated using the DNeasy blood and tissue kit (Qiagen) or phenol-chloroform extraction by resuspending in TEN (10 mM Tris, pH 8.0, 10 mM EDTA, 400 mM NaCl) with 0.1 mg/ml proteinase K and 0.2% SDS and incubating overnight at 37°C. DNA was extracted using sequential phenol-chloroform, precipitated with ethanol, and resuspended in H_2_O. Quantitative PCR was completed using primers to HCMV UL123 (5′-GCCTTCCCTAAGACCACCAAT-3′ and 5′-ATTTTCTGGGCATAAGCCATAATC-3′) and cellular TP53 (5′-TGTTCAAGACAGAAGGGCCTGACT-3′ and 5′-AAAGCAAATGGAAGTCCTGGGTGC-3′) (IDT). Quantification was completed by using Power SYBR green PCR master mix (ThermoFisher) and QuantStudio 6 Flex real-time PCR. Relative viral DNA for UL123 was normalized to cellular DNA for TP53. For RNA studies, cells were collected as described for DNA and isolated using the RNeasy kit (Qiagen). Approximately 2 μg of RNA was treated with the TURBO DNA-free kit (ThermoFisher), and cDNA was synthesized using random hexamers and SuperScript III reverse transcriptase (ThermoFisher). Relative quantities of cDNA for HCMV UL123 (5′-GCCTTCCCTAAGACCACCAAT-3′ and 5′-ATTTTCTGGGCATAAGCCATAATC-3′), UL44 (5′-GCCCGATTTCAATATGGAGGTCAG-3′ and 5′-CGGCCGAATTCTCGCTTTC-3′), and UL99 (5′-GTGTCCCATTCCCGACTCG-3′ and 5′-TTCACAACGTCCACCCACC-3′) were determined using an arbitrary standard curve, and quantities were normalized to cellular β-tubulin cDNA (5′-GCAGAAGAGGAGGATTTC-3′ and 5′-CAGTTGAGTAAGACGGCTAAGG-3′). Quantitative PCR was performed as described above.

Steady-state protein levels were measured by Western blotting. Cells were washed with PBS, collected by trypsinization, resuspended in lysis buffer (50 mM Tris-HCl, pH 8.0, 150 mM NaCl, 1% SDS) with protease inhibitor, and lysed by sonication. Protein concentrations were quantified by the Pierce bicinchoninic acid (BCA) assay kit (ThermoFisher). Proteins were resolved by SDS-PAGE using 4 to 15% (Bio-Rad) or 10% gels and transferred to a nitrocellulose membrane (GE Healthcare Life Sciences) using a semidry Trans-Blot Turbo transfer system (Bio-Rad). Membranes were blocked in 5% milk in PBS-T (phosphate-buffered saline, 0.1% Tween 20) or TBS-T (Tris-buffered saline, 0.1% Tween 20) at room temperature for 1 h before incubating with primary antibody diluted in 5% milk or bovine serum albumin (BSA) in PBS-T or TBS-T for 2 h at room temperature or overnight at 4°C. The membrane was then incubated with secondary antibody conjugated to HRP diluted in 5% milk or BSA in PBS-T or TBS-T for 2 h at room temperature. Proteins were visualized using enhanced chemiluminescence (ECL, ThermoFisher) and imaged using Bio-Rad ChemiDoc imager.

### Extracellular flux analysis.

Oxygen consumption rates (OCRs) were determined by a Seahorse XFe96 analyzer following the manufacturer’s instructions. Briefly, 30,000 (MRC-5) or 40,000 (ARPE-19) cells/well were plated in an Agilent Seahorse 96-well dish and incubated for 24 h until confluent. Cells were infected with HCMV as indicated in the figure legends. At 2 hpi, the medium was replaced with fresh medium containing treatments described in the figure legends. At 24 hpi, the medium was removed and replaced with XF base medium. Mitochondrial stress analysis was performed by sequential injections of oligomycin (1 μM), carbonyl cyanide 4-(trifluoromethoxy)phenylhydrazone (FCCP) (1 μM), and rotenone/antimycin A (1 μM) at the indicated times. Each well was normalized to micrograms of total protein determined by the Pierce BCA assay kit (ThermoFisher). Wells that did not respond following injection were removed from analysis. Each biological replicate consists of 7 to 8 technical replicates. Data were analyzed using Seahorse WAVE (Agilent) and GraphPad Prism application software.

### ATP analysis.

Cellular ATP was quantified using high-performance liquid chromatography (HPLC) as described here (79, 80). Briefly, nucleotides were extracted by perchloric acid precipitation (81) and precipitated protein was separated from supernatant through centrifugation. Supernatants were combined with solvent A (100 mM potassium phosphate buffer, 4 mM TBASH [tetrabutylammonium bisulfate, pH 6.0] diluted 64:31 with 20% methanol), and HPLC analysis was performed using a Supelcosil LC-18-T column (3 μm; 150- by 4.6-mm internal diameter) using previously established methods (80). Peaks were identified as ATP by using an ATP standard, and quantity was determined using an ATP standard curve. Precipitated protein was resuspended in 0.5 M NaOH, and concentrations were determined by the Pierce BCA assay kit (ThermoFisher).

### LC-MS/MS and metabolomics analysis.

Cells were plated subconfluently, serum starved for 24 h, and infected at an MOI of 3 IU/cells with HCMV. At 2 hpi, cells were washed with PBS, medium was replaced, and 500 μM DETA/NO or vehicle control was added. At 24 hpi, the cells were washed with ice-cold PBS, collected by scraping the dish, and snap-frozen in liquid nitrogen. Samples were analyzed by LC-MS/MS on a 1290 Infinity ultraperformance liquid chromatograph (UPLC) connected to a quadrupole time of flight mass spectrometer (Q-TOF-MS). Each sample was injected three times and subjected to reverse-phase C_18_ (positive and negative) and polar ethylene-bridged hybrid (BEH) hydrophilic interaction liquid chromatography (HILIC) (positive and negative). Raw data were normalized to total protein. Data were analyzed using Agilent MPP and MetaboAnalyst. Unpaired *t* test was used to determine significance with adjusted *P < *0.05 considered significant.

### Statistical analysis.

Experiments were analyzed using analysis of variance (ANOVA) or Student’s *t* test with multiple comparisons *post hoc* using GraphPad Prism software as indicated in the figure legends. *P < *0.05 was considered significant.
