# Supplementary figures and images for: Nitric Oxide Circumvents Virus-Mediated Metabolic Regulation during Human Cytomegalovirus Infection
Source: mBio. 2020 Dec 15;11(6):e02630-20. doi: 10.1128/mBio.02630-20 (PMC7773989; doi:10.1128/mBio.02630-20)

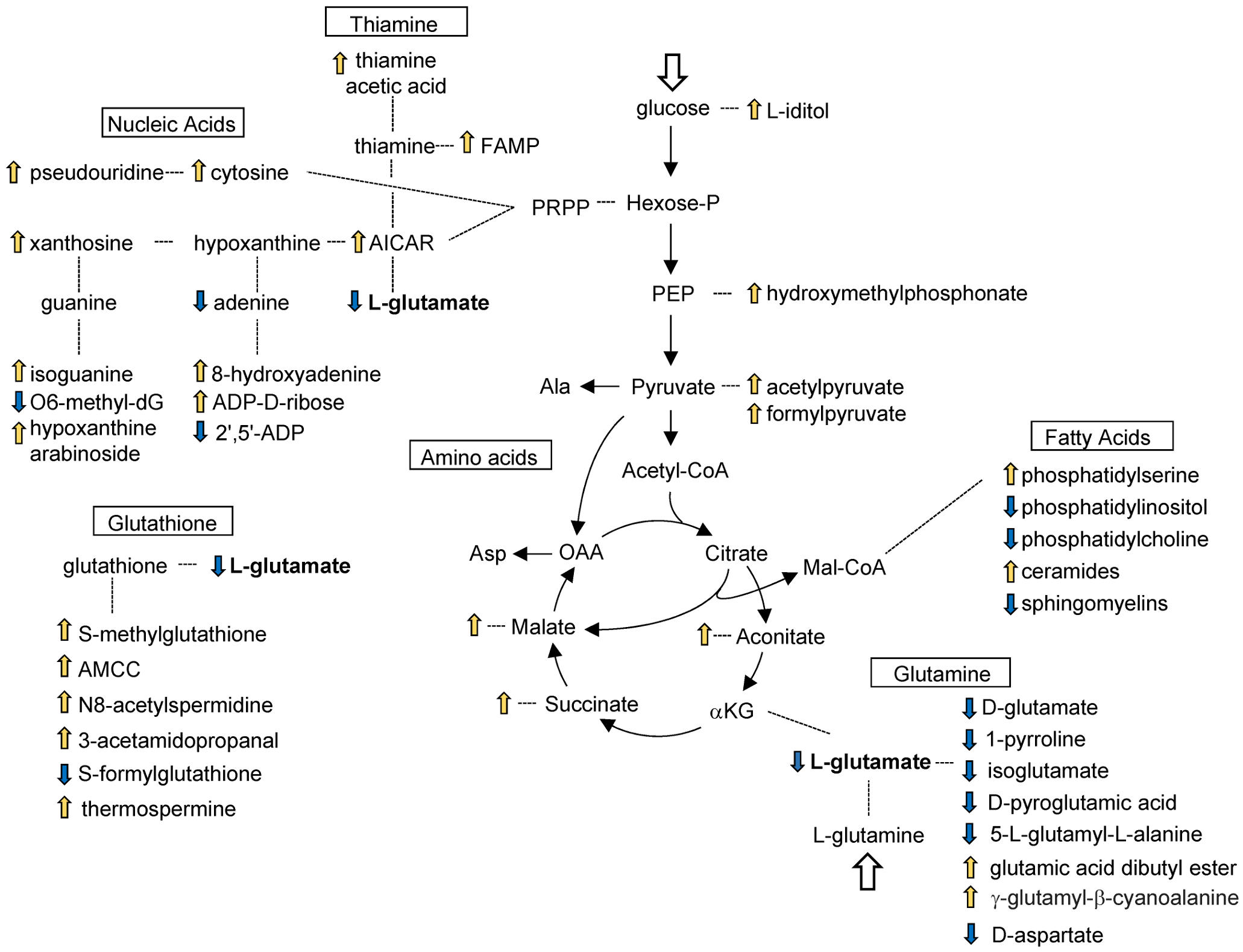

Supplement: FIG S1 [file mBio.02630-20-sf001.tif]

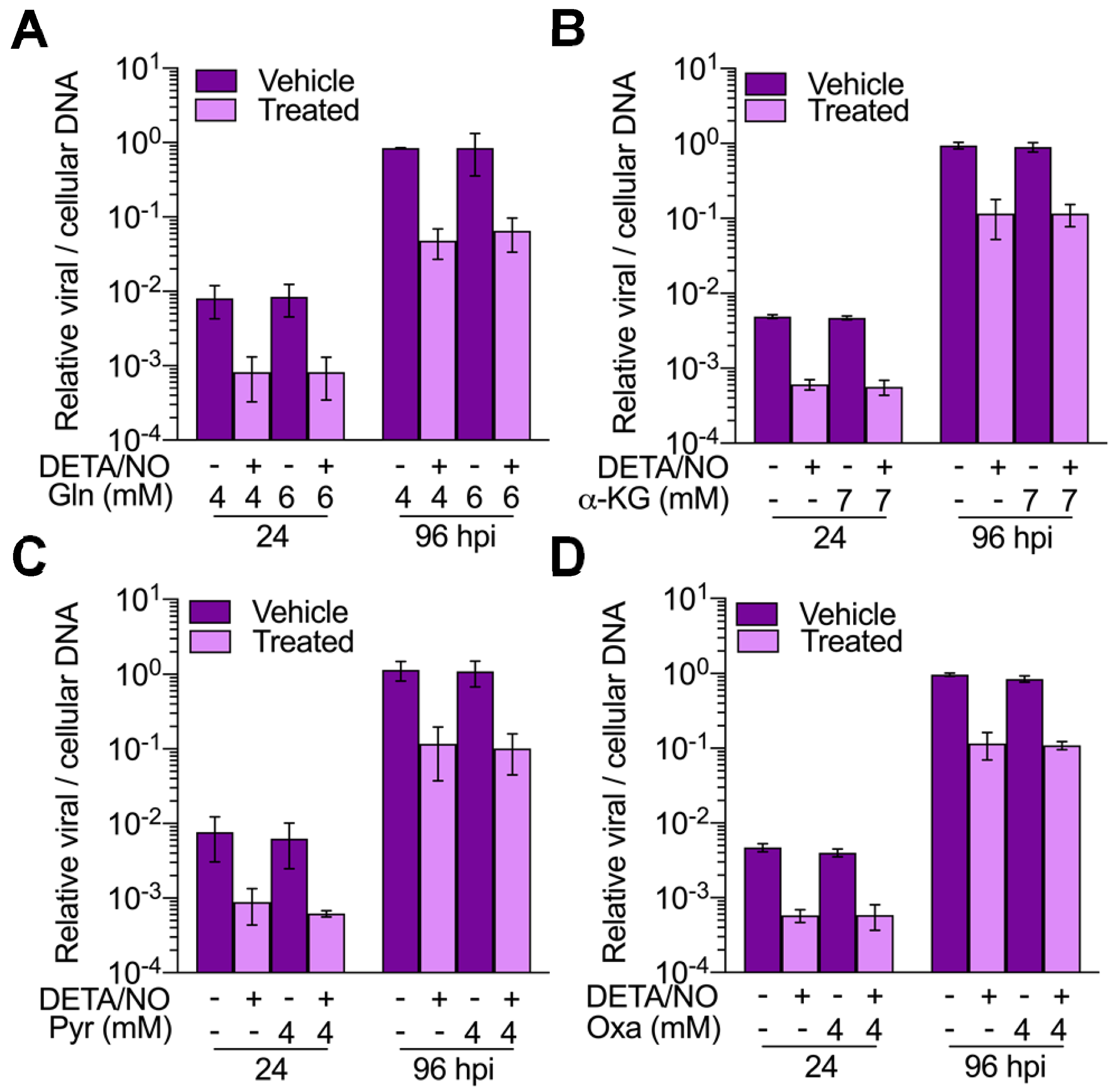

Supplement: FIG S2 [file mBio.02630-20-sf002.tif]
